# Haplotype-aware segmentation with HapASeg increases accuracy of detecting homolog-specific somatic copy number alterations

**DOI:** 10.1186/s13059-026-03971-w

**Published:** 2026-02-14

**Authors:** Oliver Priebe, Ron Solan, Conor Messer, Claudia Chu, Julian Hess, Gad Getz

**Affiliations:** 1 Broad Institute of Massachusetts Institute of Technology and Harvard, Cambridge, 02142 MA USA; 2 Krantz Center for Cancer Research and Department of Pathology, Mass General Hospital, Boston, 02115 MA USA; 3 Harvard Medical School, Boston, 02115 MA USA; 4 Present Address: Serinus Biosciences, New York, 10016 NY USA; 5 Present Address: Stanford University, Stanford, 94305 CA USA; 6 Present Address: Harvard University, Cambridge, MA 02138 USA; 7 Present Address: Predicta Biosciences, Cambridge, 02139 MA USA

**Keywords:** Cancer Genomics, SCNA estimation, FFPE

## Abstract

Somatic copy number alterations (sCNAs) drive cancer initiation, progression, resistance, and metastasis. Furthering our understanding of sCNAs requires substantially larger cohorts. Most tumors available for sequencing are preserved with formalin-fixed, paraffin-embedding (FFPE), which causes DNA cross-linking that distorts coverage profiles and challenges current sCNA estimation methods. Traditional methods denoise data using large panels of similar normal samples, which are impractical to obtain for FFPE cohorts. Here, HapASeg overcomes this limitation by leveraging haplotype phasing and unique covariates to accurately estimate sCNA segments across FFPE, fresh frozen, whole genome sequencing and whole exome sequencing sample types, outperforming current methods without requiring panel-of-normal correction.

**Supplementary Information:**

The online version contains supplementary material available at 10.1186/s13059-026-03971-w.

## Background

Copy number alterations were the first type of genetic alteration to be associated with cancerous tissue, long before the first genome was sequenced [1, 2]. Since then, the rapid advances in sequencing capabilities have furthered our understanding of sCNAs, establishing them as a major class of genomic variation and as key drivers of cancer development [3–6]. Recent progress in the field has been driven by large cancer genomic cohorts such as The Cancer Genome Atlas (TCGA) [7], Hartwig [8], and the Pan-Cancer Analysis of Whole Genomes (PCAWG) [9, 10], which were all dedicated efforts to collect fresh frozen samples. As knowledge in the field progresses, however, new cancer genomic insights will rely on analysis of larger datasets [11], including rare cancer subtypes, and/or longitudinal sampling, which are difficult to obtain as fresh frozen collections. Potentially bridging this gap, millions of patient tumor samples are routinely processed and archived by pathology laboratories around the world for morphological and immunohistochemical diagnosis each year, providing a vast repository for studies on archival samples [12–15]. The potential for such studies has largely remained unrealized due to the challenges of accurately characterizing the genomic profiles of formalin-fixed paraffin-embedded (FFPE)-preserved samples—the standard sample preservation method for archival tissue. This common preservation technique creates various artifacts across the genome, which pose technical challenges for detecting somatic variants and particularly for accurate estimation of sCNA profiles [16–18].

The challenge of characterizing sequencing data from FFPE samples stems directly from the preservation process. Formalin fixation causes hydrolytic deamination of cytosine bases to uracil, resulting in artifactual C$\,\rightarrow \,$T substitutions in sequencing data [17]. Formalin also induces cross-linking between intracellular macromolecules, which distorts fragmentation during DNA library preparation and stalls DNA polymerases during library amplification [14, 16, 19]. While the deamination artifacts are amenable to downstream correction [19, 20], the library preparation results in highly variable read depth profiles compared to fresh frozen samples, which are difficult to correct. Consequently, many established sCNA detection methods, which are often optimized for cleaner data from fresh frozen tissues, exhibit substantially reduced performance on FFPE samples. While some approaches attempt to mitigate noise by normalizing against a large panel of normal (PoN) samples, constructing such panels with appropriate tissue matching and sufficient sample-number size is often infeasible for diverse FFPE collections, limiting the applicability of these corrective strategies.

Accurate sCNA estimation is a prerequisite for numerous essential downstream analyses beyond simple aberration detection. High-resolution sCNA profiles are crucial inputs for (i) identifying driver events within amplified or deleted regions [5, 6, 21, 22], (ii) understanding tumor heterogeneity [23, 24], (iii) inferring timing of events and detecting whole-genome duplication events [25–27], and (iv) inferring tumor phylogenetic relationships to study mechanisms of resistance and metastasis [28, 29]. The inability of current methods to reliably generate high-quality sCNA calls from the most abundant sample type (FFPE) thus severely impedes progress across multiple avenues of cancer research. To overcome this critical limitation, we developed HapASeg, a novel computational method specifically designed to deliver high-accuracy, homolog-specific sCNA estimates from complex sequencing data, including FFPE samples. HapASeg leverages haplotype phasing information combined with robust noise modeling and unique covariates, enabling precise segmentation without dependence on a PoN. We demonstrate qualitatively and quantitatively that HapASeg substantially improves upon state-of-the-art methods, particularly in FFPE contexts and across varying karyotypic complexities and tumor purities. By providing reliable sCNA profiles from previously challenging samples, HapASeg may unlock the potential of vast FFPE repositories, enabling more comprehensive studies of cancer genomics, evolution, and treatment response.

In general, allele-specific sCNA detection methods use high-throughput sequencing data to detect contiguous genomic regions (i.e., segments) of the same allelic copy ratio (ACR), which is the proportion of DNA at a given locus coming from either the maternal or paternal homolog, relative to the total genomic mass of the sample. This is computed by multiplying the total copy ratio (TCR), which is the proportion of the total number of DNA molecules at the locus, and the homolog fraction (HF), which is the fraction of molecules representing each of the parental alleles. Ideally, all estimated allelic CN segments should reflect the true copy number alterations, with no spurious segments originating from noise in the sample preparation and/or sequencing process.

**Fig. 1:**
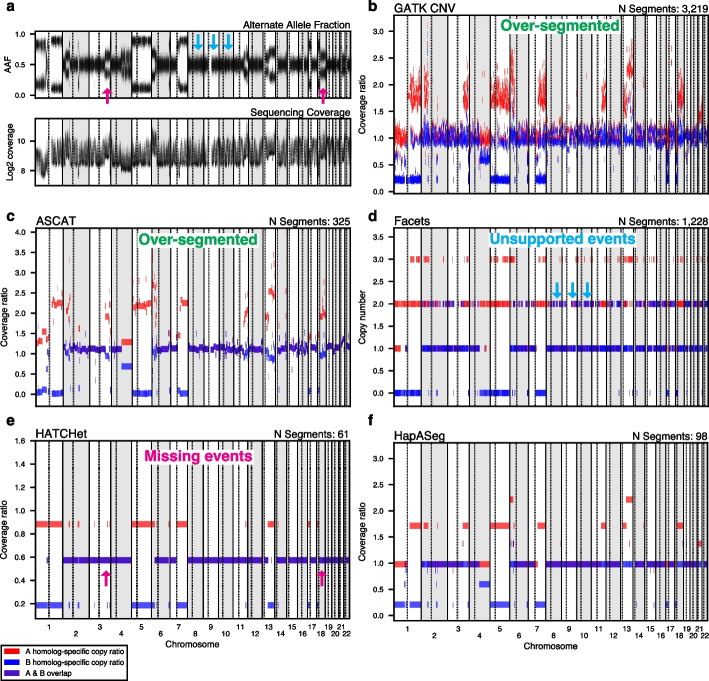
FFPE samples present difficulties for state-of-the-art somatic copy-number alteration (sCNA) detection methods. Comparison of raw data and sCNA caller results on whole genome sequencing of FFPE Richter’s transformed CLL sample CH1003. **a** Alternate allele fraction (AAF) and sequencing coverage for sample CH1003. Sequencing coverage exhibits high variance due to FFPE-driven coverage biases. Arrows highlight problematic regions in the segmentation results and mark the corresponding regions in the raw data. Blue arrows denote three likely diploid chromosomes. Pink arrows denote regions with strong evidence for sCNA events. **b**-**e** Segmentation results for four widely used sCNA methods: GATK CNV, ASCAT, Facets, and HATCHet. **f** Segmentation results for HapASeg. Minor allele segments colored in blue, major allele segments in red. The ground truth copy number profile is not known for this sample, meaning comparisons in method accuracy must be qualitative

Allelic sCNA detection methods infer TCR from the total number of tumor sequencing reads aligned to a genomic interval spanning a SNP site (sequencing coverage), and infer the HF from the fraction of non-reference reads in the tumor at germline sites heterozygous in the normal (alternate allele fraction, “AAF”). These methods segment both the AAF and coverage, combining the two into a joint allelic copy ratio segmentation. Most sCNA detection methods first segment the coverage to obtain a TCR profile, then look at AAF values within each TCR segment for evidence of allelically imbalanced segments. However, TCR segmentation is confounded by systematic fluctuations in sequencing coverage due to both biological and technical confounders. For example, early replicating regions have higher coverage than late replicating regions [30]; exceptionally GC-rich or GC-poor regions have lower coverage than regions of intermediate GC content [31]. Fluctuations exceeding the model’s expected uncertainty will produce false positive segments (typically yielding oversegmentation). Some methods [32–35] attempt to regress out fluctuations via covariates like replication timing and GC content, but cannot handle variability not predicted by these covariates. Furthermore, these methods have difficulty fitting the regression model when there is high variability in the coverage signal due to an abundance of sCNA events.

Other sCNA detection methods rely on PoN samples to empirically model these fluctuations, assuming that in a normal diploid sample any fluctuations are solely due to noise [18, 34, 36, 37]. PoN-based methods have the advantage of not needing to fit any regression parameters, since the noise profiles of the PoN samples have the same profile as the noise of the tumor. However, this approach is only applicable if the characteristics of the PoN samples are closely matched to those of the tumor sample. In particular, an effective PoN should match the sequencing technology, library type (PCR-free/PCR-plus whole genome, whole exome bait set), and sample type (fresh frozen, FFPE, cell-free DNA) of the tumor cohort under investigation. Assembling a PoN that matches all of these qualities is not always feasible, nor even always possible. For example, as of this writing, there is no publicly available large set of whole genome FFPE normal samples.

Even if an FFPE PoN existed, it is not guaranteed to be effective at denoising. Formalin crosslinks chromatin and DNA [38], hampering the ability to purify the DNA for sequencing at chromatin-rich loci, and thereby causing sequencing coverage in FFPE tumors to vary as a function of tumor-specific chromatin state, which may differ dramatically from normals in the PoN (which often consist of cells of different type). In order to effectively denoise FFPE coverage, it must be robustly regressed out without a PoN.

## Results

### sCNA detection with HapASeg

HapASeg combines several innovative techniques into a novel method for sCNA detection that delivers accurate sCNA estimates in FFPE and fresh frozen samples without the need for a PoN. First, rather than using inherently noisy TCR segmentation as its primary signal, HapASeg first performs segmentation on HF. This provides a cleaner signal, since variation in total sequencing depth affects both alleles nearly identically. Indeed, the two known factors that introduce AAF biases are exome capture bait bias and aligner bias, both of which cause negligible shifts towards the reference allele ($<3\%$, $<1\%$, respectively) that can easily be corrected for.

**Fig. 2:**
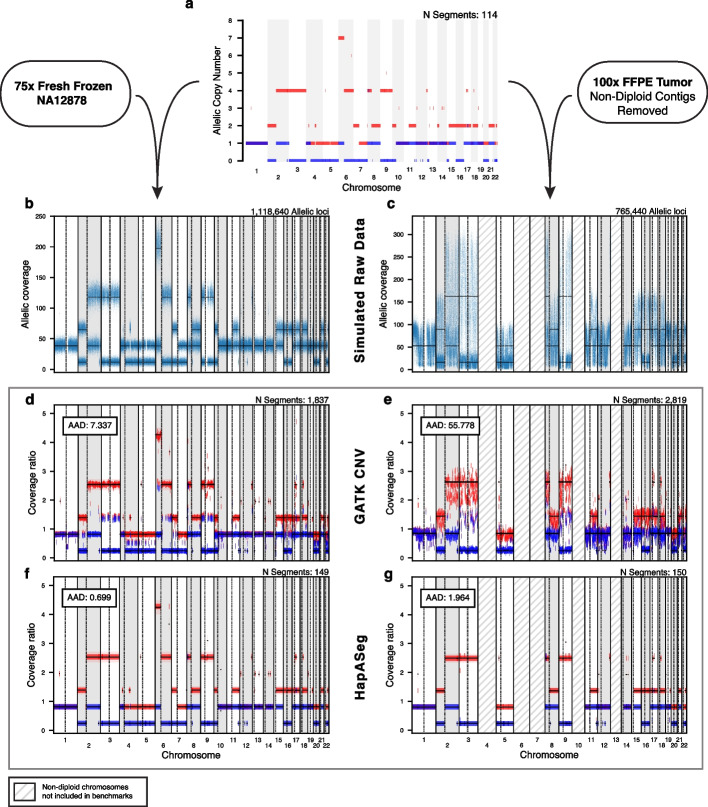
Simulating tumor samples with known karyotypes enables quantitative benchmarking of sCNA methods. **a** Simulated tumor karyotype. **b** Simulated allelic coverage derived from PCR-free whole genome sequencing of NA12878 and the simulated karyotype from (a). **c** Simulated allelic coverage derived from whole genome sequencing of Richter’s sample CH1022 and the simulated karyotype from (a). Only chromosomes that are confidently diploid were used for the simulations (non-diploid chromosomes indicated with hatches). **d**, **e** sCNA results from GATK CNV on the simulated fresh frozen and FFPE data, respectively. Segments called by the method in dark red and blue. Ground truth copy levels for each allele derived from the simulated karyotype and preservation-specific sequencing data are depicted with a black line at the mean and light red and blue boxes denoting the 95% confidence interval. Average absolute difference (AAD) between the segments estimated by the method and the ground truth in addition to the number of segments are listed above. **f**, **g** sCNA results from HapASeg on the simulated fresh frozen and FFPE data, respectively

HapASeg uses the clean HF segmentation profile to initialize the TCR segmentation. This is highly advantageous, as breakpoints of HF segments coincide almost perfectly with breakpoints of TCR segments, allowing HapASeg’s regression to robustly model coverage variability from true sCNA events (Methods). Once coverage has been adequately denoised, HapASeg performs coverage segmentation within each HF segment to infer TCR, and then combines the two signals to compute allele-specific copy ratio.

HapASeg uses a variety of covariates in this regression, many of which are well-known, e.g., GC content, replication timing, and whole-exome sequencing (WES) target lengths. To address FFPE-induced noise, HapASeg uniquely uses coverage from Formaldehyde Assisted Isolation of Regulatory Elements (FAIRE) sequencing performed on a variety ($N=37$) of normal and cancer cell lines [39]. FAIRE-seq uses formalin to crosslink DNA with bound chromatin and then sequences only accessible DNA, which emulates the same coverage irregularities observed in FFPE tumor samples. HapASeg’s robust regression combined with its unique covariates not only obviates the need for a PoN, but actually outperforms it, since FAIRE profiles include a variety of tissue types whose chromatin profiles more closely resemble those found in tumors. HapASeg is also much easier to run relative to PoN-based methods, since PoN generation can be a highly involved process at all steps, from sourcing suitable normal samples to computationally assembling their data into a panel.

Since HapASeg relies on an accurate HF segmentation, it leverages germline SNP phasing to increase its HF segmentation power. Many methods perform allelic segmentation on the raw AAF (*f*), fitting a mixture model with two clusters, where each SNP could be assigned to either a cluster at $f_\text {A}$ or a cluster at $f_\text {B} = 1 - f_\text {A}$. Therefore, such methods need to have an additional free phase ’bit’ for each SNP (i.e. whether homolog A is the reference or alternate allele). HapASeg, in contrast, uses imputed SNP phasing [40] to obtain a priori knowledge of which SNPs are likely on the same haplotype, introducing a correlation structure among the SNP phases. This reduces the uncertainty in the phases and increases the power to detect HF change points.

### Qualitative results

We first qualitatively illustrate the issues observed in current sCNA calling methods on FFPE tumor samples. We applied HapASeg and four other widely-used sCNA methods (GATK CNV [34], ASCAT [32], Facets [41], and HATCHet [42]) to a set of 16 FFPE Richter’s transformed chronic lymphocytic leukemia (CLL) samples [43, 44] of various states of degradation and tumor purities. We found that based on the signal from the raw alternate allelic fraction and coverage data (Fig. 1a), other methods’ estimates were either over-segmented (Fig. 1b, c), called sCNA events that were not supported, (Fig. 1d), or missed alterations that were evident in the raw data (Fig. 1e). We found that only HapASeg (Fig. 1f) produced reasonable sCNA estimates. These error modes were particularly distinct in samples with higher levels of degradation, but were present even in the high quality samples (see all results in Additional file 1). HapASeg’s estimates are biologically reasonable: most allelic copy ratio segments occur at uniformly spaced levels, corresponding to clonal integer copy states; segments occurring between the clonal components (e.g., chr4q, chr21q) occur at consistent distances away from the clonal integer levels, indicating they belong to the same subclone. Although this qualitatively illustrates that HapASeg performs well across a variety of real FFPE samples versus other methods that produce much noisier results, we cannot make any quantitative judgments with respect to accuracy due to the lack of a ground truth for the Richter’s data. We thus developed a robust simulation-based benchmarking platform to compare the accuracy of HapASeg to other existing methods.

**Fig. 3:**
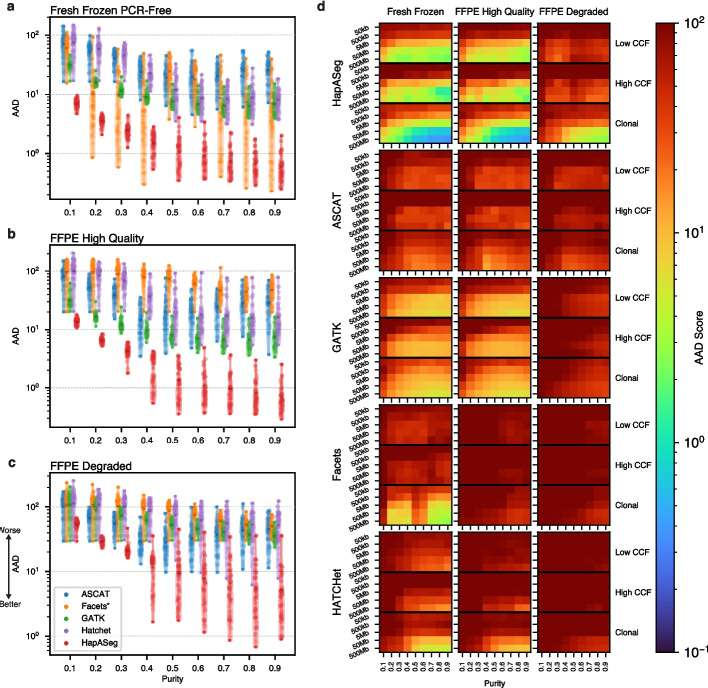
Quantitative benchmarking results. **a**-**c** Violin plots for each of the three sample preparation modalities comparing the AAD scores for the five sCNA methods analyzed across the 50 simulated tumor karyotypes in the comprehensive benchmarking panel stratified by tumor purity. Note the y-axis for AAD is in log scale and lower AAD scores denote higher accuracy. Dashes within violin plots denote the mean and range. **d** AAD heatmap examines the accuracy of the sCNA methods stratified by purity, ground truth event length and the cancer cell fraction (CCF) of the simulated sCNA segments (clonal: CCF=1, low: CCF < 0.7, high: CCF $\ge$ 0.7). *Facets only returns absolute copy number results, which are used here for computing AAD scores

### Quantitative benchmarking

Many sCNA detection methods have been developed, but few attempts have been made to systematically assess their performance across the range of karyotype complexities, sample preparation methods, and sequencing modalities encountered in real world settings. This is primarily due to the absence of a set of tumor samples with known ground truth copy number profiles at all possible event sizes and subclonal fractions. Thus, benchmarking approaches that use real tumor samples can either (i) qualitatively assess relative differences between methods (Sequenza [33], CNV Radar [37], SEQC-II [45]), or (ii) only assess performance on a limited subset of the karyotypes assayed by orthogonal means (e.g., FISH [32], CNV Radar [37]; or ddPCR [46]). On the other hand, using simulated tumors with real noise profiles to benchmark against yields much more comprehensive ground truth data across karyotypes of arbitrary complexity. This allows us not only to absolutely quantify a method’s performance at every single genomic locus, but also to stratify our assessment based on event type (e.g., subclonal focal events). Previous approaches using simulated tumors have failed to robustly assess sCNA detection accurately due to using overly simplistic models in their simulations, which fail to capture the noise and biases of real sequencing data (HATCHet [42], CloneHD [47]), or have been limited in the number and types of events that they could simulate (TITAN [35], Falcon [46], ReMiXT [48]). To better evaluate the accuracy of HapASeg with respect to other state-of-the-art methods, we developed a new simulation-based benchmarking approach, called CNV-Suite, which addresses the previous shortcomings.

CNV-Suite can simulate any karyotype, defined by a set of allele and clone-specific copy number events (e.g., gains, losses, whole-genome doubling, loss-of-heterozygosity, chromothripsis). As mentioned in the Introduction, there are two primary signals that allele-specific sCNA detection algorithms leverage from sequencing data: the tumor HF at germline heterozygous variant sites, and the TCR inferred from tumor sequencing coverage. CNV-Suite simulates both of these components by taking known diploid samples (both fresh frozen and FFPE) and directly scaling their real allele counts and read coverage according to the synthetic tumor karyotype and desired purity (Additional file 2: Fig. S1; Methods). Crucially, this approach maintains the intrinsic noise of the sequencing data, making the simulated tumors essentially indistinguishable from real tumors with the same sample characteristics as their underlying diploid progenitors. The simulated tumor allele count and read coverage data can be passed directly to each method under evaluation, at which point its results can be directly compared to the ground truth. Figure 2 illustrates the CNV-suite benchmarking workflow: A simulated tumor karyotpe (Fig. 2a) is used to generate simulated raw data from chosen reference fresh frozen (Fig. 2b) and FFPE (Fig. 2c) tumor samples. The simulated raw data files are then passed to sCNA calling methods, and the results are compared against the ground truth karyotype (Fig. 2d-g).

We used CNV-Suite to create a comprehensive benchmarking panel composed of 50 synthetic tumor karyotypes, each consisting of up to three subclones and a randomized selection of copy number events. Event lengths were randomly sampled from the reported $\sim 1/\mathrm{length}$ distribution of sCNAs found in real cancers [5] (Methods). The number of events as well as the clone for each event were also chosen randomly in each sample, resulting in a total of 4,637 simulated sCNA events across a range of simulated karyotype complexities (Additional file 3). We then simulated tumor coverage/allele counts for each karyotype using three different underlying progenitor samples — fresh frozen, high-quality FFPE, and highly degraded FFPE, at purities from 10%−90% in 10% increments, for a total of 50 x 3 x 9 = 1,350 simulated tumors.

To measure the accuracy of the predicted sCNA profile for each method to the ground truth, we compute the average absolute distance (AAD) between the allelic copy ratio for each predicted sCNA segment against the allelic copy ratio in the ground truth karyotype, with each distance weighted by the segment’s length. Since the units and magnitude of the output segment copy ratios vary among the methods, and also vary within each method across purities, we rescale (and shift) the predicted profile such that it achieves the lowest (i.e., best) AAD score (Additional file 2: Fig. S2, Methods). Estimated sCNA segments and absolute difference scores for each method are tabulated for each simulated profile at purity 0.7 in Additional file 4.

This approach allowed us to dissect the accuracy of each model for each sample type by ground truth event length and clonal cell fraction (CCF). HapASeg was able to consistently outperform all other methods for both fresh frozen and FFPE samples across the entire range of purities, event sizes, and clonal fractions, often by an order of magnitude (Fig. 3a-d). Some methods, such as FACETS, aggressively smooth the segmentation profile, which can result in better scores for certain simulated samples, but is detrimental on average. HapASeg particularly excelled relative to other methods on the high-quality FFPE-derived samples. This success will facilitate future studies leveraging the vast archives of paraffin slides that have, until now, remained largely inaccessible for precise genomic characterization. We also benchmarked simulated WES samples using the same synthetic tumor panel. We found that HapASeg outperformed other methods, particularly in clonal sCNAs over 50Mb (Additional file 2: Fig. S3). All benchmarked methods performed poorly on WES tumors for sCNAs less than 50Mb, suggesting that current methods approach the limit of detection at this length on WES samples and investigators should turn to whole genome analyses for high-resolution estimates.

To further assess the accuracy of HapASeg across other tissue types, we performed an additional quantitative benchmarking analysis using a cohort of the nine cases from TCGA with fresh frozen and FFPE tumor samples derived from the same biopsy and characterized with WGS (Methods). We ran each sCNA method on the tumor samples and quantified their accuracy using the matched fresh frozen sample results as ground truth (Additional file 2: Fig. S4, Methods). While samples from the same biopsy may differ due to tumor heterogeneity and therefore only serve as imperfect sources of true copy number, the results support HapASeg’s improved performance on FFPE samples across tissue types.

## Discussion

Somatic copy number alterations (sCNA) are a major class of genetic variation in cancer. Accurate detection of sCNAs from sequencing data is essential for understanding the mechanisms of therapy resistance and metastasis in cancer. Existing tools, surveyed above, have been successfully deployed to estimate sCNAs in high quality samples or in settings that require lower resolution estimates. However, progress has been hampered by the difficulty of accurately estimating sCNAs in FFPE samples, which comprise the majority of archived clinical tissue. To address this, we developed HapASeg, a novel computational method we describe herein that leverages germline haplotype phasing information and unique genomic covariates to accurately estimate allele-specific sCNAs without relying on a correction based on a PoN, which are difficult to compile from FFPE samples. Our findings show that HapASeg markedly outperforms current state-of-the-art methods in accuracy across various sample types (FFPE, fresh frozen, WGS, WES) and karyotypic complexities, validated both on challenging Richter’s transformed CLL FFPE samples and on 1,800 synthetic samples using a robust simulation-based benchmarking platform (that is also part of this manuscript).

This work represents several major advances for the field: 1) novel methodology; we introduce a fundamentally new approach for detecting somatic CN alterations (sCNAs) that integrates haplotype phasing and PoN-free noise regression using unique covariates (e.g., FAIRE-seq), achieving significant improvements in accuracy across all samples types. 2) unlocking analysis of FFPE diagnostic and archived samples; HapASeg demonstrates superior performance on FFPE samples, overcoming a major technical barrier and making diagnostic samples and vast archives of clinically annotated tumor samples accessible for high-resolution genomic studies of cancer evolution (including improved phylogenetic reconstruction), resistance, and metastasis. 3) rigorous benchmarking resource (CNV-Suite): We provide an open source simulation suite that generates realistic tumor sequencing data, allowing for comprehensive and quantitative benchmarking of sCNA detection methods across diverse complexities and sample types. HapASeg is designed to be easily executed on local computer or on high performance cloud computing platforms, as described in the open code repository. The overall runtime of the method can take several hours on a standard 8 core machine for whole genome sequenced samples; however several steps of the method are amenable to additional parallelization if compute resources are available.

While HapASeg represents a significant methodological advancement, some limitations of the approach may inform future directions. The ability for HapASeg to correct noise in sequencing coverage from FFPE preservation depends on the degree of similarity between the tumor coverage profile and the 37 coverage profiles in the FAIRE panel. The existing FAIRE panel has been effective in denoising the wide variety of tissue types analyzed by our group for other research projects. In the future, FAIRE sequencing of additional tumor types could improve the method. Additionally, HapASeg does not attempt to infer or filter germline copy number polymorphisms (CNP)s by default. Germline CNPs will likely confound HapASeg’s sCNA estimates, and users are encouraged to pass a bed file of regions to exclude from sCNA analysis for samples with known CNPs. Future versions of HapASeg may perform germline CNA estimation on the normal sample to better account for CNPs.

**Fig. 4:**
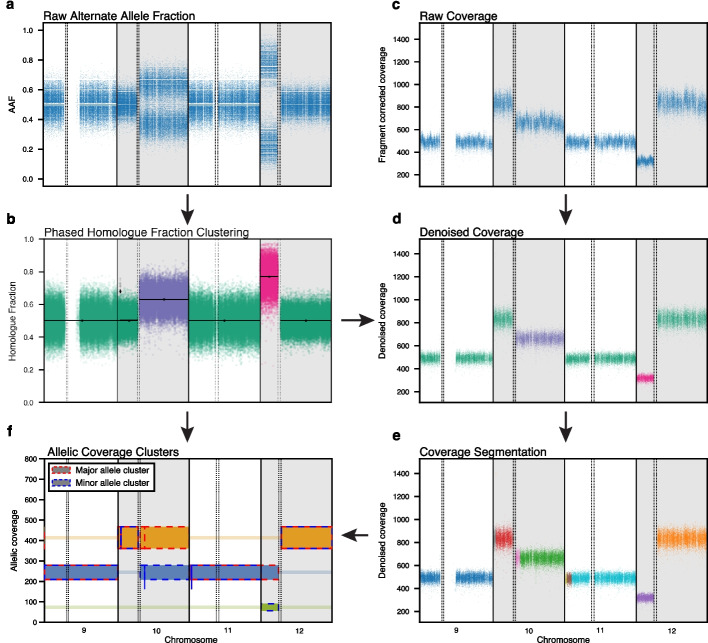
HapASeg method schematic. **a** Alternate allele fraction is computed at confidently heterozygous SNP sites. **b** Phased homolog fraction is segmented and clustered across the genome. SNP sites are colored by homolog fraction cluster. **c** Raw fragment length corrected coverage is computed in genomic intervals. **d** Initial coverage segments are created using the breakpoints inferred by the homolog fraction clusters and coverage covariates are applied to regress out known biases in coverage. The color of each point corresponds to the homolog fraction cluster that it overlaps. **e** corrected coverage within each initial coverage segment is further segmented using the log-normal poisson model. **f** Homolog fraction segments and coverage segments are combined to create the final allele-specific coverage segments. Each allelic coverage cluster is depicted with colored rectangles, with height corresponding to the 95% confidence interval

## Conclusions

In this work, we present HapASeg, a novel homolog-specific sCNA prediction method that leverages genomic covariates and haplotype phasing to accurately estimate sCNA events without requiring a PoN. We demonstrate HapASeg’s superior sCNA calling ability both qualitatively, using FFPE Richter’s transformed CLL tumors, and quantitatively, via CNV-Suite, an extensive open source benchmarking platform that we developed for comparing sCNA callers with simulated tumors. In our simulation experiments, we found that HapASeg sCNA calls were more accurate than the four state-of-the-art methods we benchmarked against, often by an order of magnitude. We observe that the improved sCNA estimates from HapASeg were consistent across sample purity, event lengths, and sequencing modality. The improved accuracy of HapASeg in FFPE samples is particularly poised to unlock the vast supply of archived tumor samples, which previously had unusable sCNA profiles. These newly unlocked FFPE samples, combined with the increased accuracy of sCNA calls in other sample types, will offer new insight into the mechanisms of metastasis and resistance in cancer.

## Methods

### Homolog fraction (HF) segmentation

#### SNP processing

For each SNP coordinate in a reference panel of phased germline SNPs (e.g., 1000 Genomes [49]), we extract the read count pileups from the tumor/normal alignments for each allele at that coordinate in the panel. These read counts can be used to visualize raw AAF as in Fig. 4a. We use MuTect [50] for this purpose, since it is efficient, has a robust set of read filters, and most importantly, counts alleles on the fragment level (i.e., read pair level), rather than the single read level. This is critical for sample types with short fragments where reads in a pair may overlap (e.g., FFPE or cfDNA); double counting alleles in overlapping reads will distort downstream results.

We genotype whether a site is heterozygous in the normal sample via the following procedure. Let $n_{\text {Nalt}_i}$ and $n_{\text {Nref}_i}$ be the normal alternate and reference allele counts in the pileup for the *i*th SNP site. Then, assuming the fraction of alternate reads *f* is beta distributed, i.e.

$$\begin{aligned} f\sim \mathrm{Beta}(n_{\text {Nalt}_i} + 1, n_{\text {Nref}_i} + 1), \end{aligned}$$

SNP site *i* is considered heterozygous if the absolute posterior log odds ratio follows

$$\begin{aligned} |\ln {\mathrm{Pr}(\,f \ge 0.5\,)} - \ln {\mathrm{Pr}(\,f \le 0.5\,)}| < 2.5. \end{aligned}$$

The threshold of 2.5 was empirically chosen because it yields a 90% true positive rate for heterozygous (het) site genotyping (assuming uniformly distributed Q30 error rates), irrespective of sequencing coverage. Because we only consider SNPs in a curated reference panel (that thus reside in genomic regions with much lower error rates), the actual false positive rate is vanishingly small.

In order to properly impute phasing, we also must call homozygous SNPs in the normal, since phasing imputation relies on proper genotyping for all SNPs in the sample. Any SNP in the panel confidently close to $f = 1$ is considered homozygous, according to the criterion $\mathrm{Pr}(f \ge 0.95)> 0.7$.

We impute phasing using Eagle2 [40], given the SNP genotypes and the reference panel. Eagle2 assigns each allele in each heterozygous allele (het) locus to either the A or B haplotype. Let $n_{\text {Talt}_i}$ and $n_{\text {Tref}_i}$ be the tumor alt/ref read counts for the *i*th SNP site, and $a_i,b_i$ be the corresponding haplotype-specific tumor read counts for SNP *i*. If the alternate allele is phased to the A haplotype, then $a_i = n_{\text {Talt}_i}$, and $b_i = n_{\text {Tref}_i}$; if the alternate allele is phased to the B haplotype, then $a_i = n_{\text {Tref}_i}$, and $b_i = n_{\text {Talt}_i}$. We define the homolog fraction (HF) of SNP *i* as $f_i = a_i/(a_i + b_i)$.

#### Segmentation MCMC

Index SNPs are ordered by genomic position from $i = 1\dots N_s$, and let $j=1\dots N$ denote the allelic segment. Let the interval set $\mathcal {I}_j=[s_j, e_j)$ comprise all SNP indices falling between $s_j$ and $e_j - 1$. Let $f_j$ be the homolog fraction of $\mathcal {I}_j$. Assuming only aleatoric uncertainty (i.e., $f_j$ is a random variable whose stochasticity comes only from binomial sampling), the likelihood of $f_j$ for interval $\mathcal {I}_j$ is

$$\begin{aligned} \mathcal{L}(f_j|\mathcal{I}_j) &= \prod_{i \in \mathcal{I}_j} f_j^{a_i} (1 - f_j)^{b_i}\\ &= f_j^{\sum_{i\in\mathcal{I}_j} a_i}(1-f_j)^{\sum_{i\in\mathcal{I}_j} b_i} \\ &\equiv f_j^{A_j}(1-f_j)^{B_j} \end{aligned}$$

where $A_j$ and $B_j$ are the total read counts respectively assigned to homologs $\text {A}$ and $\text {B}$ within interval $\mathcal {I}_j$. Marginalizing out $f_j$ yields the marginal likelihood of the interval,

$$\begin{aligned} \mathcal {L}(\mathcal {I}_j)= & \int _0^1 df_j\,f_j^{A_j}(1-f_j)^{B_j}\\= & \beta (A_j + 1, B_j + 1). \end{aligned}$$

Finding an optimal segmentation is equivalent to finding an optimal partitioning of all SNPs, i.e. the optimal set of intervals $\{\mathcal {I}_1 = [0, e_1), \mathcal {I}_2 = [s_2, e_2), \dots , \mathcal {I}_N = [s_N, e_N)\}$, where $s_j=e_{j-1} \; \forall \, 1<j \le N$. The likelihood of a given segmentation is the product of the marginal likelihoods of each interval,

$$\begin{aligned} \mathcal {L}(\mathcal {I}_1,\dots ,\mathcal {I}_N)= & \prod _{j=1}^N\mathcal {L}(\mathcal {I}_j)\\= & \prod _{j=1}^N\beta (A_j + 1, B_j + 1). \end{aligned}$$

Unfortunately, for $N_s$ total SNPs, the total number of possible partitions is $2^{N_s - 1}$, which is intractably large for ${\sim }10^6$ SNPs in a whole genome, or ${\sim }10^4$ SNPs in a whole exome.

Luckily, the space of partitions is amenable to efficient sampling via Markov Chain Monte Carlo (MCMC) simulation, yielding high likelihood solutions. Initially, each SNP belongs to its own segment. At random, we pick two adjacent segments and probabilistically merge them according to the Metropolis criterion:

1 $$\begin{aligned} \mathrm{Pr}(\underbrace{[s_j,e_j),[s_{j+1},e_{j+1})}_{S}\rightarrow \underbrace{[s_j,e_{j+1})}_{S^*}) = \min \left\{ 1,\frac{\mathcal {L}(S^*)q(S|S^*)}{\mathcal {L}(S)q(S^*|S)}\right\} , \end{aligned}$$

where marginal likelihoods are

2 $$\begin{aligned} \mathcal {L}(S^*)= & \beta \bigg(\underbrace{\sum\nolimits_{\{i|i\in [s_j, e_{j+1})\}}a_i}_{A_{[s_j,e_{j+1})}}+1,\underbrace{\sum\nolimits_{\{i|i\in [s_j, e_{j+1})\}}b_i}_{B_{[s_j,e_{j+1})}}+1\bigg)\end{aligned}$$

3 $$\begin{aligned} \mathcal {L}(S)= & \beta (A_{[s_j,e_j)}+1,B_{[s_j,e_j)}+1)\times \beta (A_{[s_{j+1},e_{j+1})}+1,B_{[s_{j+1},e_{j+1})}+1). \end{aligned}$$

We can also split segments of length $>1$. Rather than picking a breakpoint within the segment at random, we probabilistically tailor our proposal distribution. For each SNP $k\in [s_j,e_j)$, we compute

4 $$\begin{aligned} \mathcal {L}_j(k) = \beta (A_{[s_j,k)}+1,B_{[s_j,k)}+1)\times \beta (A_{[k,e_j)}+1,B_{[k,e_j)}+1), \end{aligned}$$

and propose picking *k* as the breakpoint with probability $p_j(k)=\mathcal {L}_j(k)/\sum _k \mathcal {L}_j(k)$. Our proposal distributions are

$$\begin{aligned} q(S|S^*) = \frac{p_j(k)}{N} \qquad q(S^*|S) = \frac{1}{N-1} \end{aligned}$$

where *N* is the total number of segments at the current MCMC iteration.

The overall MCMC procedure is:

1. Initialize each SNP to belong to its own segment.
2. With equal probability, pick whether to attempt a merge or split this iteration.
3. If we choose to merge, uniformly pick segment *j* at random from 1 to $N-1$. Then, we probabilistically merge it with segment $j+1$ with probability defined in (1).
4. If we choose to split, uniformly pick *j* at random from 1 to *N*, propose a breakpoint within *j* via (4), and accept the proposal with probability defined as the reciprocal of (1). Proposing a breakpoint at the end of the segment is equivalent to not accepting any proposal at all and leaving the chain as-is.
5. We run the chain until it has burned-in, which we infer by detecting whether the overall likelihood oscillates around a maximum, rather than monotonically increasing. In practice, we check if the mean overall likelihood difference between each of the last 1000 iterations is less than or equal to zero. We then run the chain up to 20,000 iterations, stopping early if the chain converges to a segmentation with a maximal overall likelihood value that does not change for 1000 consecutive iterations.
6. The maximum likelihood segmentation from the chain post–burn-in is used in subsequent processing.

The MCMC is extremely fast, since each iteration only involves computing partial sums in (2), (3), and (4). The MCMC is also efficiently parallelized: the most compute-intensive phase is at the beginning when each SNP belongs to its own segment. Blocks of adjacent SNPs are burned-in in parallel, which are then concatenated post–burn-in to sample the final segmentation posterior. During this step, each chromosome arm can be run in parallel, since we do not expect reliable phasing across the centromere.

#### Reference bias correction

The alternate allelic fraction at a SNP site can be biased from an overrepresentation of reference reads, especially in capture-based whole exome sequencing, since baits only target the reference genome and thus preferentially bind fragments supporting the reference. Small amounts of reference bias may also manifest in whole genome sequencing due to aligners’ slightly diminished ability to map reads containing mismatches.

We correct for reference bias empirically. Recall that a SNP assigned to haplotype A with homolog fraction $f = a/(a + b)$ means that *a* is the read count of the alternate allele, and *b* is the read count of the reference allele; an SNP assigned to haplotype B has *a* as the reference count and *b* as the alternate count. Thus, for SNPs in segment $S_j$, the overall homolog fraction across all SNPs assigned to A is distributed

$$\begin{aligned} f_{\text {A},j}\sim & \mathrm{Beta}(\sum\nolimits_{i\in \text {A}\cap S_j} a_i + 1, \sum\nolimits_{i\in \text {A}\cap S_j} b_i + 1)\\\sim & \mathrm{Beta}(\sum\nolimits_{i\in \text {A}\cap S_j} n_{\text {Talt}_i} + 1, \sum\nolimits_{i\in \text {A}\cap S_j} n_{\text {Tref}_i} + 1). \end{aligned}$$

Similarly, the homolog fraction across all SNPs assigned to haplotype B is

$$\begin{aligned} f_{\text {B},j}\sim & \mathrm{Beta}(\sum\nolimits_{i\in \text {B}\cap S_j} a_i + 1, \sum\nolimits_{i\in \text {B}\cap S_j} b_i + 1)\\\sim & \mathrm{Beta}(\sum\nolimits_{i\in \text {B}\cap S_j} n_{\text {Tref}_i} + 1, \sum\nolimits_{i\in \text {B}\cap S_j} n_{\text {Talt}_i} + 1). \end{aligned}$$

In the absence of reference bias, if we had infinite SNPs assigned to A and B, the absolute difference between $f_A$ and $f_B$ would approach zero; $\lim _{|A|\rightarrow \infty ,|B|\rightarrow \infty }|f_\text {A} - f_\text {B}| = 0$. In the presence of reference bias, all reference allele counts are scaled by some constant factor $r_b<1$,

$$\begin{aligned} f_{\text {A},j}(r_b)\sim & \mathrm{Beta}(\sum\nolimits_{i\in \text {A}\cap S_j} n_{\text {Talt}_i} + 1, r_b\sum\nolimits_{i\in \text {A}\cap S_j} n_{\text {Tref}_i} + 1)\\ f_{\text {B},j}(r_b)\sim & \mathrm{Beta}(r_b\sum\nolimits_{i\in \text {B}\cap S_j} n_{\text {Tref}_i} + 1, \sum\nolimits_{i\in \text {B}\cap S_j} n_{\text {Talt}_i} + 1). \end{aligned}$$

Immediately after burn-in, we perform a grid search over $r_b\in [0.7,1]$, and minimize the mean absolute difference across segments, weighted by the number of SNPs per segment, $n(S_j)$:

$$\begin{aligned} r_b = \underset{r_b}{\arg \min }\frac{\sum _j|f_{\text {A},j}(r_b) - f_{\text {B},j}(r_b)|\times n(S_j)}{\sum _j n(S_j)}. \end{aligned}$$

We compute the absolute difference via Monte Carlo (i.e., sample the beta distributions, and take the mean difference between samples). We then scale all alternate allele counts by $r_b$, and perform all subsequent allelic segmentation operations with scaled reference allele counts. In our experience, reference bias in whole genome samples is minimal ($\sim 0.99$), since it is only due to aligner bias. It is higher in whole exome samples ($\sim 0.93$) due to capture bait bias.

### Allelic segmentation refinement

Imputed phasing cannot yield perfectly consistent homolog assignments from telomere-to-telomere, due to the fact that the genotype of an individual being imputed will be a combination of the haplotypes present in the reference panel. Thus, at best, homolog assignments will be consistent relative only to a contiguous haplotype block within the imputed individual, and may even experience orientation switches within a haplotype block due to meiotic recombination events. SNP haplotype assignments are generally consistent relative to each other within an average of 14.4Mb, or about 14 centimorgans (see Appendix 5 for details). This means that a long HF segment will typically be split into several components alternating between *f* and $1 - f$. We must correct for this phase orientation switching in post-processing.

To address this, we refine our optimum initial segmentation, jointly performing phase orientation correction and clustering of non-adjacent segments using a Dirichlet process (DP) MCMC. Clustering non-adjacent segments improves sensitivity by increasing the probability that the model produces extremely focal segments supported by few SNPs if their homolog fraction is consistent with other non-adjacent segments. We refer to this procedure as the Allelic Dirichlet Process, or ADP.

We initialize each non-phase corrected segment into its own cluster, yielding clusters indexed as $\textbf{K}=\{1,\dots ,N\}$. We formulate the MCMC as a Gibbs sampler: at each MCMC iteration, we choose a segment *S*, remove it from the cluster it is currently a part of, and probabilistically join a currently existing cluster, or open a new cluster, conditioned on the configuration of all other segments. Let $A_S$ and $B_S$ correspond to the total segmental read counts of homologs A and B, and $\tilde{A}_k = \sum _{s\in C_k} A_s$ and $\tilde{B}_k = \sum _{s\in C_k} B_s$ be the total read counts of A and B across all segments assigned to cluster $k\in \textbf{K}$. We also include pseudo counts $\mathfrak {a}, \mathfrak {b}$ for read counts of homolog A and B, respectively, to stabilize the marginal likelihoods at low read counts. In practice we set $\mathfrak {a}=\mathfrak {b}=10$.

The probability that *S* joins $C_k$ is proportional to the overall likelihood of the system, which is the product of (i) the marginal likelihood of *S* combined with $C_k$,

5 $$\begin{aligned} \mathcal {L}(S\cup C_k) = \beta (\tilde{A}_k + A_S + \mathfrak {a} + 1, \tilde{B}_k + B_S + \mathfrak {b} + 1) \end{aligned}$$

and (ii) the marginal likelihood of every other cluster $\{C_\kappa \mid \kappa \ne k\}$,

$$\begin{aligned} \mathcal {L}(\{C_\kappa \mid \kappa \ne k\}) = \prod _{\kappa \ne k} \mathcal {L}(C_\kappa ) = \prod _{\kappa \ne k}\beta (\tilde{A}_\kappa + \mathfrak {a} + 1, \tilde{B}_\kappa + \mathfrak {b} + 1). \end{aligned}$$

This yields

$$\begin{aligned} p(S\cup C_k)\propto & \mathcal {L}(S\cup C_k)\mathcal {L}(\{C_\kappa \mid \kappa \ne k\})p_\text {DP}(S\cup C_k)\\\propto & \frac{\mathcal {L}(S\cup C_k)\mathcal {L}(\{C_\kappa \mid \kappa \in \textbf{K}\})}{\mathcal {L}(C_k)}p_\text {DP}(S\cup C_k), \end{aligned}$$

since $\prod _{\kappa \ne k}\mathcal {L}(C_\kappa ) = \prod _{\kappa \in \textbf{K}}\mathcal {L}(C_\kappa )/\mathcal {L}(C_k)$. The term in the numerator does not depend on *k* and thus is a constant that can be canceled, leaving us with

$$\begin{aligned} p(S\cup C_k)\propto & \frac{\mathcal {L}(S\cup C_k)}{\mathcal {L}(C_k)}p_\text {DP}(S\cup C_k)\\\propto & p(S|C_k)p_\text {DP}(S\cup C_k), \end{aligned}$$

where $p_\text {DP}(S\cup C_k)$ is the DP prior on $C_k$, which is based on the number of SNPs in both $C_k$ and *S*. This is an extension of the standard DP prior, since it accounts not only for the number of entities in the cluster being joined but also the number of entities being moved. See Appendix 2 for details.

*S* can also create a new cluster ($k = |\textbf{K}|+1$) using the above formalism, by setting $\tilde{A}_k$ and $\tilde{B}_k$ to 0, i.e.,

$$\begin{aligned} \mathcal {L}(C_{|\textbf{K}|+1} \cup S)= & \beta (A_S + \mathfrak {a} + 1, B_S + \mathfrak {b} + 1)\\ \mathcal {L}(C_{|\textbf{K}|+1})= & \beta (\mathfrak {a} + 1, \mathfrak {b} + 1). \end{aligned}$$

#### Phasing correction

To address phase orientation switching events that are not resolved by imputation, we also allow segments to probabilistically flip their orientation. Without this correction, we would observe segments at alternating HF levels *f* and $1 - f$ at mis-phased switch sites. Phase correction is accomplished by considering a likelihood of phase orientation during MCMC steps. We adopt the convention of orienting each segment’s homolog fraction to $f\ge 0.5$; in other words, the copy number assigned to the A haplotype should always be greater or equal to the number of copies assigned to the B haplotype. We impose this convention by asserting that the probability that a segment’s phasing orientation is properly oriented is the probability that the total number of alternate reads assigned to the A haplotype is higher than the total number of alternate reads assigned to the B haplotype. We sum the alt and ref counts across all SNPs in segment *S*, segregated by whether the SNP is assigned to A or B:

$$\begin{aligned} \begin{array}{ll} A_\text {alt} = \sum \limits _{i\in \text {A}} n_{i,\text {alt}}\qquad & B_\text {alt} = \sum \limits _{i\in \text {B}} n_{i,\text {alt}}\\ A_\text {ref} = \sum \limits _{i\in \text {A}} n_{i,\text {ref}}\qquad & B_\text {ref} = \sum \limits _{i\in \text {B}} n_{i,\text {ref}}. \end{array} \end{aligned}$$

The haplotype-specific alternate allele fractions are beta distributed,

$$\begin{aligned} f_A\sim & \mathrm{Beta}(A_\text {alt} + \mathfrak {a} + 1, A_\text {ref} + \mathfrak {b} + 1)\\ f_B\sim & \mathrm{Beta}(B_\text {alt} + \mathfrak {a} + 1, B_\text {ref} + \mathfrak {b} + 1) \end{aligned}$$

and thus the probability that a segment is oriented “properly” is given by the difference

$$\begin{aligned} p(\phi ):=\mathrm{Pr}[\,f_A - f_B> 0\,]. \end{aligned}$$

When computing the marginal likelihood of joining an existing cluster, we jointly consider the phasing orientation by computing *both* phasing orientations of the segment being moved in (5), i.e.,

$$\begin{aligned} \mathcal {L}(C_k \cup S|\phi = 0)= & \beta (\tilde{A}_k + A_S + \mathfrak {a} + 1, \tilde{B}_k + B_S + \mathfrak {b} + 1)\\ \mathcal {L}(C_k \cup S|\phi = 1)= & \beta (\tilde{A}_k + B_S + \mathfrak {a} + 1, \tilde{B}_k + A_S + \mathfrak {b} + 1), \end{aligned}$$

yielding the joint probability

$$\begin{aligned} p(S,k,\phi ) = p(S|C_k,\phi )p(\phi ) p(C_k\cup S). \end{aligned}$$

At each MCMC iteration, the new cluster assignment *k* and phasing orientation $\phi$ for segment *S* are drawn from the joint posterior

6 $$\begin{aligned} p(k,\phi |S) = \frac{p(S|C_k,\phi )p(\phi ) p(C_k\cup S)}{\sum _{k\in \textbf{K}}\sum _{\phi \in \{0,1\}}p(S|C_k,\phi )p(\phi ) p(C_k\cup S)}. \end{aligned}$$

#### Joint segmentation and clustering

If two adjacent segments join the same cluster, they are subsequently considered as a single segment. This greatly increases the efficiency of the DP to reach an equilibrium state, but can also lead the MCMC down an irreversible path. To mitigate this, we also allow segments to be probabilistically split, in a manner identical to the splitting procedure of the initial segmentation MCMC (4). In this case, we enumerate the likelihoods of all possible split points and probabilistically draw accordingly, and then randomly choose either the right or left side of the split segment as a candidate to reassign in the DP.

Finally, to avoid hyper-segmentation in the DP, we penalize for placing adjacent segments in different DP clusters. It is possible that, by chance, a genomic region’s homolog fraction is slightly more similar to a distant segment than to its immediate neighbors, and would be assigned to the same cluster as the distant segment, even though it truly belongs in the same segment with its neighbors. Clustering that is naïve to local segmentation would thus tend to oversegment. To mitigate this tendency to oversegment, we add a penalty term to the ADP that encourages the segment being moved to be in the same cluster as its neighbors.

Given segment $S_i$ being moved, and its immediate neighbors $S_{i-1}$ and $S_{i+1}$ respectively assigned to clusters $k_{i-1}$ and $k_{i+1}$, we compute the following likelihoods:

$$\begin{aligned} p(S|k_{i-1},k_{i+1},\phi = 0) \propto \left\{ \begin{array}{ll} \beta (A_{i-1} + A_i + A_{i+1} + \mathfrak {a} + 1,& k_{i-1} = k_i = k_{i+1}\\ B_{i-1} + B_i + B_{i+1} + \mathfrak {b} + 1)& \\ \beta (A_{i-1} + A_i + \mathfrak {a} + 1, B_{i-1} + B_i + \mathfrak {b} + 1)& k_{i-1} = k_i \ne k_{i+1}\\ \quad \times \beta (A_{i+1} + \mathfrak {a} + 1, B_{i+1} + \mathfrak {b} + 1)& \\ \beta (A_{i+1} + A_i + \mathfrak {a} + 1, B_{i+1} + B_i + \mathfrak {b} + 1)& k_{i-1} \ne k_i = k_{i+1}\\ \quad \times \beta (A_{i-1} + \mathfrak {a} + 1, B_{i-1} + \mathfrak {b} + 1)& \\ \beta (A_i + \mathfrak {a} + 1, B_i + \mathfrak {b} + 1)& k_{i-1} \ne k_i \ne k_{i+1}\\ \quad \times \beta (A_{i-1} + \mathfrak {a} + 1, B_{i-1} + \mathfrak {b} + 1)& \\ \quad \times \beta (A_{i+1} + \mathfrak {a} + 1, B_{i+1} + \mathfrak {b} + 1)& \\ \end{array}\right. \end{aligned}$$

(Analogous likelihoods are computed for $\phi = 1$, by swapping $A_i$ and $B_i$ in each expression.) The appropriate term is added to (6) depending on the upstream and downstream segments’ cluster assignments.

Adding the locality penalty, the joint Gibbs sampler posterior becomes

$$\begin{aligned} p(k,\phi |S) = \frac{p(S|C_k,\phi )p(S|k_{i-1},k_{i+1},\phi )p(\phi ) p(C_k\cup S)}{\sum _{k\in \textbf{K}}\sum _{\phi \in \{0,1\}}p(S|C_k,\phi )p(S|k_{i-1},k_{i+1},\phi )p(\phi ) p(C_k\cup S)}. \end{aligned}$$

As with the initial segmentation MCMC, we use the ADP as a stochastic optimizer, running it until it reaches an equilibrium and then selecting the maximum likelihood MCMC sample.

The final refined allelic segmentation comprises contiguous regions of SNPs assigned to the same ADP cluster at the maximum likelihood sample (Fig. 4b). Formally, given SNPs ordered by genomic position indexed from $i = 1\dots N$, we denote the SNP-specific cluster assignments as $k_1\dots k_{N}$. We also denote the *j*th refined segment as

7 $$\begin{aligned} S_j = \{i\mid i\in [s_j, e_j), k_i = \kappa _j\forall i\},\qquad s_j = e_{j-1} \; \; \forall j \ge 1 . \end{aligned}$$

### Total copy ratio segmentation

The final result of the ADP yields an optimal segmentation based on homolog fraction (HF) alone. However, this is only half of the information needed for inferring allelic copy ratio, and in turn, absolute allelic copy number. We also need the total copy ratio (TCR) at each SNP. This is inferred from the overall genomic coverage in intervals spanning the SNPs—uniform windows for whole genomes, individual exons for whole exomes—which, in the absence of systematic coverage biases, would be proportional to the total amount of genomic mass within each interval, and thus the TCR of that interval.

However, because systematic biases do exist, we cannot simply segment the coverage *de novo* to obtain a TCR profile that is independent of the HF segmentation. Instead, we leverage the fact that HF segments frequently correspond perfectly to TCR segments, since the mapping between HF and TCR is nearly unique at the segment level; it is rare that an HF segment comprises multiple distinct TCR states (see Appendix 1). Nonetheless, it is sometimes possible, so we use the HF segmentation to establish a strong prior foundation on the TCR segment intervals.

We leverage this prior by initializing all TCR segments in 1:1 correspondence with HF segments. This lets us fit an initial regression model to remove coverage noise that correlate with known genomic covariates. We then perform an additional round of segmentation on the residuals of the regression, capturing TCR segments within HF segments that have degenerate HF$\rightarrow$TCR mappings.

#### Coverage processing

We calculate the fragment length-normalized coverage for each window $w_i = (\mathfrak {s}_i, \mathfrak {e}_i)$, where $\mathfrak {s}_i$ and $\mathfrak {e}_i$ are genomic start and end coordinates, respectively, for interval *i* (Fig. 4c). While other CNV callers simply compute coverage as the number of sequencing fragments in the window, this fails to account for the fact that different genomic windows may have different fragment length distributions. Total genomic mass of a window is proportional to the total number of sequencing bases in the window, not the total number of fragments, since longer fragments contribute more genomic mass than shorter fragments. Thus, for each $w_i$, we sum the lengths of all fragments overlapping the window and divide by the average fragment length in the entire sample to obtain fragment length-normalized coverage. Coverage window intervals can be defined arbitrarily, but for the analyses described herein, we use uniform two kilobase windows for WGS data, and exon capture intervals for WES data.

#### Coverage denoising

We assign each coverage window that overlaps a SNP to its corresponding HF segment, as defined in (7). For whole genomes, we do not consider coverage windows that do not overlap SNPs, since assigning an allelic copy ratio state to a window requires joint knowledge of its HF state and its TCR state. There are more coverage bins than SNPs (the SNP reference panel yields between $5\times 10^5$ and $10^6$ SNPs per genome, depending on how closely the patient’s ethnicity matches members of the reference panel; there are approximately $1.6\times 10^6$ 2kb bins in a human genome), so it is possible for a highly focal event to not overlap any SNPs, but still be distinguishable via coverage alone. We do not consider such events, since their allele-specific copy number states would be ambiguous, even if their TCRs are resolvable. However, whole exomes only have ${\approx }3\times 10^4$ SNPs, which we found insufficient to robustly regress out the higher coverage noise relative to whole genomes. For exomes, we therefore also consider coverage windows within 10kb of a SNP, and impute their HF state based on the neighboring SNP.

Given the *j*th allelic segment $S_j$ containing SNPs $j_1,\dots ,j_{N_j}$, we denote the corresponding coverage segment containing overlapping windows as $\Omega _j = \{w_{i(j_1)},\dots ,w_{i(j_{N_j})}\}$. Note that $|\Omega _j|\le |S_j|$, since some coverage windows may contain multiple SNPs. Let $\vec c_i$ be covariates for $w_i$. We fit a Poisson generalized linear model (GLM) across all coverage windows:

$$\begin{aligned} w_i|\vec c_i, j_i \sim \mathrm{Pois}(\lambda _i|\vec c_i, j_i)\qquad \log \lambda _i|\vec c_i, j_i = \mu _{j_i} + \vec \beta \cdot \vec c_i, \end{aligned}$$

whose model likelihood is

$$\begin{aligned} \mathcal {L}(\{\mu _j\},\vec \beta | \{w_i\},\{\vec c_i\},\{j_i\}) = \prod _i \mathrm{Pois}(w_i;\exp (\mu _{j_i} + \vec \beta \cdot \vec c_i)). \end{aligned}$$

This likelihood is log-convex and can thus be globally optimized by Newton-Raphson to obtain maximum likelihood parameters $\{\hat{\mu }_j\}$ and $\hat{\vec {\beta }}$. Fitting coverage segment-specific means $\{\mu _j\}$ allows the slope parameter $\vec \beta$ to only reflect coverage variability due to covariates, and not due to true copy alterations. An accurate estimate of $\vec \beta$ is critical for subsequent coverage segmentation. The covariates used are described in Table 1.

**Table 1:** Coverage denoising covariate descriptions

GC content	Regions of both high and low GC content have depressed coverage due to PCR inefficiencies during library prep [31]. High GC content makes it difficult to melt DNA duplexes, and low GC content makes it difficult to anneal primers. We found that the relationship between coverage and GC content is close to quadratic, and thus we add two covariates for GC content (fraction of C or G bases in the window, $f_\text {GC}$, and $f_\text {GC}^2$) to accommodate a second-order model
Replication timing	Early replicating regions have higher coverage [30]. We use replication timing tracks at the 50 kb resolution, derived from Hansen et al. [51]
FAIRE-seq	As the name implies, Formaldehyde Assisted Isolation of Regulatory Elements (FAIRE) is an assay originally developed to identify regions of open chromatin that uses formalin to crosslink DNA to bound chromatin, and then purifies and sequences the non-crosslinked DNA. FAIRE-seq was performed in 37 cell lines comprising various normal tissues and cancers [39]. We found that a linear combination of their coverage profiles can closely match the coverage spikes due to open chromatin in FFPE tumor samples
Matched normal coverage	Leveraging the matched normal coverage as a covariate can remove incorrect signal from SNPs in non-diploid balanced germline copy number events. SNPs in imbalanced regions of amplifications in the normal will not be called as heterozygous SNPs, since their allelic fractions will be significantly distinct from 50%, which is not the case for balanced germline copy-number events. If the tumor and normal share similar sequencing characteristics, this can further suppress systematic noise not captured by the other covariates
Capture target length	Since coverage windows in whole exomes are the exon capture intervals, window-specific coverage will be proportional to the length of the exon

For certain capture kits for WES, one can add a panel-of-normals as covariates, since the variability in coverage cannot be fully explained by the variables above. We find that this regression approach substantially reduces the noise in tumor coverage profiles for the samples we have tested (Fig. 4d).

### Coverage segmentation

Once we have an accurate estimate of $\hat{\vec {\beta }}$ to denoise coverage, we perform an additional round of segmentation within each allelic segment. To compute the likelihood of sub-segments, we use a log-normal Poisson (LNP) GLM

$$\begin{aligned} w_i|\vec c_i \sim \mathrm{Pois}(\lambda _i|\vec c_i)\qquad \log \lambda _i|\vec c_i = \mu + \hat{\vec {\beta }}\cdot \vec c_i + \sigma \epsilon _i\qquad \epsilon _i\sim \mathcal {N}(0, 1), \end{aligned}$$

which is robust to the observed overdispersion of coverage levels. The LNP has advantages in flexibility and interpretability when compared to other overdispersed count models such as the negative binomial (also called Gamma-Poisson).

Given some allelic segment with corresponding coverage segment $\Omega _j = \{w_{\mathfrak {s}_j},\dots w_{\mathfrak {e}_j+1}\}$ spanning the interval $[\mathfrak {s}_j, \mathfrak {e}_j)$, the marginal likelihood of $\Omega _j$ containing no breakpoints is

8 $$\begin{aligned} \mathcal {L}([\mathfrak {s}_j,\mathfrak {e}_j)):= & \int _{M,\Sigma }d\mu \,d\sigma \,\mathcal {L}(\mu ,\sigma |\{w_i\mid j\in [\mathfrak {s}_j, \mathfrak {e}_j)\},\{\vec c_i\mid j\in [\mathfrak {s}_j, \mathfrak {e}_j)\},\hat{\vec {\beta }})\nonumber \\= & \int _{M,\Sigma }d\mu \,d\sigma \,\prod _{i=\mathfrak {s}_j}^{\mathfrak {e}_j - 1}\int _{-\infty }^\infty d\epsilon _i\,\mathrm{Pois}(w_i;\exp (\mu + \hat{\vec {\beta }}\cdot \vec c_i + \sigma \epsilon _i))\times \mathcal {N}(\epsilon _i;0,1). \end{aligned}$$

Marginal likelihoods for arbitrary disjoint sub-segmentations with breakpoints at $\{b_1,\dots ,b_N\}$, $\mathfrak {s}_j< b_1< \dots< b_i< b_{i+1}< \dots < \mathfrak {e}_j$ are given by

$$\begin{aligned} \mathcal {L}(\{b_1,\dots ,b_N\}) = \mathcal {L}([\mathfrak {s}_j,b_1))\times \dots \times \mathcal {L}([b_{i},b_{i+1}))\times \mathcal {L}([b_{i+1},b_{i+2}))\times \dots \times \mathcal {L}([b_N,\mathfrak {e}_j)), \end{aligned}$$

with the limits of the product in (8) modified accordingly for each term.

We sample from the distribution of possible segmentations via Gibbs sampling. Initially, let all coverage windows within $S_j$ belong to the same segment. In each step of the Gibbs sampler, we first choose a segment $\Omega _j$ at random. Then, we elect to perform a split or a merge operation on $\Omega _j$ with equal probability. For a split operation, we compute the marginal likelihood of splitting $\Omega _j$ at each possible split point $b_i$, with likelihood $\mathcal {L}(\texttt {split}_{b_i}) = \mathcal {L}([\mathfrak {s}_j,b_i)) \times \mathcal {L}([b_i,\mathfrak {e}_j))$ conditioned on all other segments remaining unchanged. We also compute the likelihood of the existing segment $\mathcal {L}(\Omega _j) = \mathcal {L}([\mathfrak {s}_j, \mathfrak {e}_j))$ and we probabilistically chose a split at $b_i$ according to the normalized likelihood

$$\begin{aligned} \mathrm{Pr}(\texttt {split}_{b_i})= \frac{\mathcal {L}(\texttt {split}_{b_i})}{\sum _{\ell =\mathfrak {s_j + 1}}^{\mathfrak {e_j - 1}}{ \mathcal {L}(\texttt {split}_{b_\ell }) + \mathcal {L}(\Omega _j)}}. \end{aligned}$$

We also allow $\mathcal {L}(\Omega _j)$ to be chosen, which designates no change for the segment.

Alternatively, if a join operation is elected, we compute the likelihood of joining $\Omega _j$ with its neighbor $\Omega _{j-1}$ and accept with probability

$$\begin{aligned} \mathrm{Pr}(\texttt {join}_j) = \frac{\mathcal {L}(\mathfrak {s}_{j-1}, \mathfrak {e}_j)}{\mathcal {L}(\mathfrak {s}_{j-1}, \mathfrak {e}_j) + \mathcal {L}(\Omega _{j-1}) + \mathcal {L}(\Omega _{j})} \end{aligned}$$

Note that if $j < 2$, the iteration is completed without altering the segmentation.

Computing the marginal likelihood of a segment is computationally expensive, so we limit the proposed breakpoints using the split heuristic described in the following section. Also, note that the likelihood of a single coverage bin is undefined. To avoid this issue, we restrict segments to contain at least two coverage windows. This yields a theoretical limit of detection of 4kb for WGS, and two adjacent exons for WES. As with allelic MCMC chains, we use the coverage segmentation MCMC as a stochastic optimizer, running it until it reaches an equilibrium and then selecting the maximum likelihood MCMC sample (Fig. 4e).

#### Choosing segment split points

Because coverage segments can potentially comprise thousands of windows, enumerating the entire set of all possible split points would be too slow. Instead, to limit the search space, we probabilistically determine split candidates with a heuristic that convolves two adjacent boxcar kernels (“change kernel”) of varying widths $W=\{10, 50, 100, 250\}$ across all coverage windows in the segment being split, and take the absolute difference $d_{\mathit{W,i}}$ of both convolutions at each window *i*.

For each *W*, we store any position *i* whose difference $d_{\mathit{W,i}}$ exceeds the 98th percentile of all differences for that kernel width. This defines an initial set of candidate split points. However, the optimal split points as found by the change kernel heuristic may not correspond to the best split points based on their marginal likelihood. Hence, we scan around each split point, in both directions (up to 5 windows for large segments; 15 for small), computing the split marginal likelihood of neighboring points until the split marginal likelihood falls below 5 natural log units below the maximal split marginal likelihood, or the window limit is reached.

This procedure outputs a subset of most likely split points $b_1, \dots , b_N$, with associated marginal likelihoods $\mathcal {L}_1 ,\dots ,\mathcal {L}_N$. We probabilistically pick from these proposals as described in the previous section.

#### Marginal likelihood computation

Both integrals in (8) are analytically intractable. To compute the inner integral

$$\begin{aligned} I_i := \int _{-\infty }^\infty d\epsilon _i\,\mathrm{Pois}(w_i;\exp (\mu + \hat{\vec {\beta }}\cdot \vec c_i + \sigma \epsilon _i))\times \mathcal {N}(\epsilon _i;0,1) \end{aligned}$$

we use Gaussian quadrature restricted to a neighborhood around the peak of the integrand. Although in principle Hermite quadrature matches the domain of integration, we observed in many cases that the integrand was too sharply peaked for traditionally selected Hermite polynomial roots to accurately approximate the integral. We were able to robustly compute the integral by instead using 25 Legendre roots rescaled within the interval $[\hat{c} - 6\hat{r}, \hat{c} + 6\hat{r}]$, where $\hat{c}$ is the real component of the root of the integrand, and $\hat{r}$ is the $\sigma$ corresponding to a Gaussian distribution fit to the curvature of the integrand at $\hat{c}$ using Laplace’s method.

To compute the outer integral

$$\begin{aligned} \int _{M,\Sigma }d\mu \,d\sigma \,\prod _{i=s_j}^{e_j - 1} I_i \end{aligned}$$

we use Laplace’s approximation.

#### LNP prior

To both (i) avoid over-segmentation due to noise and (ii) make the LNP optimization more robust, we impose a prior on its parameters. We choose the normal inverse gamma distribution, since it is not conjugate to the LNP, but it is conjugate to the normal and Poisson distributions. For LNP parameters $\mu$ and $\sigma$, we have normal and inverse gamma priors, respectively,

$$\begin{aligned} \mu \, & |\, \sigma , \, \mu _{prior}, \, \lambda \sim \mathcal {N}(\mu _{prior}, \sigma ^2 / \lambda ) \\ \sigma ^2 \, & | \, \alpha , \, \beta \sim \Gamma ^{-1}(\alpha , \beta ) \end{aligned}$$

where $\Gamma ^{-1}$ denotes the inverse gamma distribution. From this, we derive the joint normal inverse gamma PDF

$$\begin{aligned} \mathcal {N}\Gamma ^{-1}(\mu , \sigma ) = \frac{\sqrt{\lambda }}{\sigma \sqrt{2 \pi }}\left( \frac{1}{\sigma ^2}\right) ^{\alpha + 1} \exp \left( - \frac{2 \beta + \lambda ( \mu - \mu _{prior})^2}{2 \sigma ^2} \right) \end{aligned}$$

We find empirically that setting $\alpha = 10^{-5}$, $\beta = 4\times 10^{-3}$ works well across the benchmarking and tumor samples we have tested. We also make the prior on $\mu$ uninformative by setting $\lambda = 10^{-10}$ and $\mu _{prior} = \sum _{w \in \Omega _j}{\log (w)}/|\Omega _j|$, i.e., the mean of the log coverage windows.

### Allelic coverage clustering

At this stage, we have found optimal HF and TCR segmentations. Each coverage window $w_i$ is jointly associated with an HF segment index $h_i$ and TCR segment index $t_i$; denote this tuple $u_i = (h_i, t_i)$. Our initial set of allelic copy ratio (ACR) segments comprises contiguous sets of coverage windows with identical tuples. Formally, the *j*th ACR segment $\Xi _j$ comprises all coverage windows such that

$$\begin{aligned} \Xi _j = \{i\mid u_i = u_{i-1}\forall i\}. \end{aligned}$$

The overall HF $\alpha _j$ of $\Xi _j$ is given by the beta distribution

$$\begin{aligned} \alpha _j|\{a_i\}_j,\{b_i\}_j\sim \mathrm{Beta}(\sum\nolimits_{i\in \Xi _j} a_i + 1, \sum\nolimits_{i\in \Xi _j} b_i + 1) \end{aligned}$$

where $a_i$ and $b_i$ are the total read counts for SNPs within coverage window $w_i$ assigned to the A and B homologs, respectively. Likewise, the overall TCR $\tau _j$ of $\Xi _j$ is given by the log-normal Poisson distribution

$$\begin{aligned} \tau _j|\{w_i\}_j,\{\vec c_i\}_j,\hat{\vec {\beta }}\sim \mathrm{LNP}(\mu _j, \sigma _j|\{w_i\mid i\in \Xi _j\}, \{\vec c_i\mid i\in \Xi _j\}, \hat{\vec {\beta }}) \end{aligned}$$

where $\mu _j$ and $\sigma _j$ are found by fitting the LNP regression described in Coverage segmentation section to the coverage bins and covariates assigned to $\Xi _j$.

The product of random variables $\alpha _j$ and $\tau _j$ yields two homolog-specific ACR (HSACR) distributions for $\Xi _j$, $\xi _{j,\text {A}}$ for the A homolog and $\xi _{j,\text {B}}$ for the B homolog:

9 $$\begin{aligned} \xi _{j,\text {A}}\sim \alpha _j\times \tau _j\qquad \xi _{j,\text {B}}\sim (1 - \alpha _j)\times \tau _j. \end{aligned}$$

Since human genomes only have two homologs, the homolog fraction of the B allele is by definition $1 - \alpha _j$. For $n_\Xi$ total ACR segments, there are thus $2n_\Xi$ total HSACR segments. In regions of allelic balance ($\hat{\alpha }_j = 0.5$), the HSACR segments overlap.

These HSACR segments are suitable for use by downstream tools (e.g., ABSOLUTE [25], PhylogicNDT [29]), and they can be further refined by combining segments with identical levels of HSACR that have distinct HF and TCR levels. For example, a triploid region with two copies of homolog A and one copy of homolog B has HF and TCR levels distinct from a region of copy-neutral loss-of-heterozygosity (two copies of homolog A, zero copies of homolog B), even though both share a common level of ACR for homolog A (two copies in both regions). Highly focal regions supported by few coverage windows/SNPs have intrinsically high measurement uncertainty; by combining the focal regions with other regions of the same HSACR, we increase our ability to definitively assign ACR states to these focal regions. To do this, we employ an additional round of Dirichlet process clustering, which we call the allelic coverage Dirichlet process (ACDP) (Fig. 4f).

#### ACDP model

For each pair of the HSACR segments $\xi _{j,\text {A}}$ and $\xi _{j,\text {B}}$, we estimate the distribution of the product of the random variables in (9) via a Monte Carlo simulation, since there is no closed-form expression for the product of beta and LNP random variables. Each ACR segment consists of $n_j = |\xi _j|$ coverage bins, so we draw $n_j$ samples from the corresponding beta and LNP distributions and compute the product of each draw. Denote these sets of samples

$$\begin{aligned} \tilde{\boldsymbol{\xi }}_{j,\text {A}}= & \{\tilde{\alpha }_i\tilde{\tau }_i\mid \tilde{\alpha }_i\sim \alpha _j,\tilde{\tau }_i\sim \tau _j,i\in \{1\dots n_j\}\}\\ \tilde{\boldsymbol{\xi }}_{j,\text {B}}= & \{(1 - \tilde{\alpha }_i)\tilde{\tau }_i\mid \tilde{\alpha }_i\sim \alpha _j,\tilde{\tau }_i\sim \tau _j,i\in \{1\dots n_j\}\}. \end{aligned}$$

We found that the distribution of the product of random variables is nicely approximated by a normal distribution, allowing us to fit a Dirichlet process Gaussian clustering model across all HSACR segments. The likelihood of each HSACR segment is given by a normal distribution,

10 $$\begin{aligned} \mathcal {L}(\mu ,\lambda |\underbrace{\tilde{\boldsymbol{\xi }}_{j,\text {A}}}_{\tilde{\textbf{X}}_j}) = \prod _{i=1}^{n_j}\mathcal {N}(\underbrace{\tilde{\alpha }_i\tilde{\tau }_i}_{\tilde{x}_i};\mu ,\lambda )\qquad \mathcal {L}(\mu ,\lambda |\tilde{\boldsymbol{\xi }}_{j,\text {B}}) = \prod _{i=1}^{n_j}\mathcal {N}((1 - \tilde{\alpha }_i)\tilde{\tau }_i;\mu ,\lambda ) \end{aligned}$$

with a normal-gamma prior on the mean and precision parameters,

$$\begin{aligned} p(\mu ,\lambda ;\mu _0, \kappa _0, \alpha _0, \beta _0)= & \mathcal {N}\mathcal {G}(\mu , \lambda ;\mu _0, \kappa _0, \alpha _0, \beta _0) \\= & \mathcal {N}(\mu ;\mu _0, (\kappa _0 \lambda )^{-1})\mathcal {G}(\lambda ;\alpha _0, \text {rate}=\beta _0) \end{aligned}$$

where $\mathcal {G}$ denotes the gamma distribution. We have prior-weighted likelihood

11 $$\begin{aligned} p(\tilde{\textbf{X}}_j|\mu ,\lambda ,\underbrace{\mu _0, \kappa _0, \alpha _0, \beta _0}_{\mathcal {H}}) = \left[ \prod _{i=1}^{n_j}\mathcal {N}(\tilde{x}_i;\mu ,\lambda )\right] \mathcal {N}\mathcal {G}(\mu , \lambda ;\mathcal {H}), \end{aligned}$$

whose marginal likelihood has closed form,

12 $$\begin{aligned} p(\tilde{\textbf{X}}_j|\mathcal {H})= & \int _{M,\Lambda } d\mu \,d\lambda \,p(\tilde{\textbf{X}}_j|\mu ,\lambda ,\mathcal {H})\nonumber \\= & \frac{\Gamma (\alpha _n)}{\Gamma (\alpha _0)}\frac{\beta _0^{\alpha _n}}{\beta _n^{\alpha _0}}\sqrt{\frac{\kappa _0}{\kappa _n}\frac{1}{(2\pi )^{n_j}}} \end{aligned}$$

where $\Gamma$ denotes the gamma function, with

$$\begin{aligned} \begin{array}{ll} \mu _n = \frac{\kappa _0\mu _0 + n_j\langle {\tilde{\textbf{X}}_j}\rangle }{\kappa _0 + \langle {\tilde{\textbf{X}}_j}\rangle } \qquad & \kappa _n = \kappa _0 + n_j \\ \alpha _n = \alpha _0 + n_j/2\qquad & \beta _n = \beta _0 + \frac{1}{2} \sum \limits _{i=1}^{n_j}(x_i - \langle {\tilde{\textbf{X}}_j}\rangle )^2 + \frac{\kappa _0 n_j (\langle {\tilde{\textbf{X}}_j}\rangle - \mu _0)^2}{2(\kappa _0 + n_j)}. \end{array} \end{aligned}$$

#### Hyperparameters

We employ a prior to account for some latent noise in the system not reflected in the data likelihood. The normal-gamma distribution is conjugate to the joint posterior on $(\mu ,\lambda )$, making hyperparameter interpretation intuitive—the prior distribution is equivalent to a likelihood with $\kappa _0$ observations of $\mu$ with sample mean $\mu _0$, with precision estimated from $2\alpha$ observations with sample mean $\mu _0$ and sum of squared deviations $2\beta$. We empirically set $\alpha _0$ to the average number of data points $\langle {n_j}\rangle$ across all HSACR segments and $\beta _0 = \sum _{i=1}^n(x_i - \bar{x})^2/2$ to the average sum of squared deviations for each set of segment data points. Since we lack prior knowledge for what the mean value of a cluster should be, we leave the prior on $\mu$ uninformative.

#### MCMC procedure

The closed-form nature of the marginal likelihood for a set of HSACR segments makes optimizing the ACDP clustering model amenable to MCMC sampling in a manner similar to the allelic clustering model defined in Allelic segmentation refinement section. We initialize each HSACR segment in its own cluster, yielding clusters indexed as $\textbf{K}= \{1\dots 2n_\Xi \}$. At each MCMC step, we pick an HSACR segment at random, unassign it from its current cluster, and probabilistically join it with an existing cluster, or open a new cluster.

The probability that an HSACR segment $H = \tilde{\boldsymbol{\xi }}$ joins cluster $C_k$ is proportional to

$$\begin{aligned} p(H\cup C_k|\mathcal {H})\propto & \frac{\mathcal {L}(H\cup C_k)}{\mathcal {L}(C_k)}p_\text {DP}(H\cup C_k)\\\equiv & p(H|C_k,\mathcal {H})p_\text {DP}(H\cup C_k) \end{aligned}$$

where

$$\begin{aligned} \mathcal {L}(H\cup C_k)= & p(\tilde{\boldsymbol{\xi }}\cup \{\tilde{x}_i\mid i \in C_k\}|\mathcal {H})\\ \mathcal {L}(C_k)= & p(\{\tilde{x}_i\mid i \in C_k\}|\mathcal {H}), \end{aligned}$$

with marginal likelihoods defined in (12), HSACR sets $\tilde{\boldsymbol{\xi }}$ and $\{\tilde{x}_i\}$ defined in (10), and DP prior $p_\text {DP}(H\cup C_k)$ defined in Appendix 2. *H* can also create a new cluster by setting $C_k = \emptyset$, which makes $p(H|\emptyset ,\mathcal {H})$ fall back on the normal-gamma prior in (11).

To speed up the MCMC, we also propose merging entire clusters. At random, we pick a cluster $C_j$, unassign all segments therein, and compute probabilities and underlying marginal likelihoods of joining every other cluster (in a manner similar to above),

13 $$\begin{aligned} p(C_j\cup C_k) & \propto p(C_j|C_k,\mathcal {H})p_\text {DP}(C_j\cup C_k) \nonumber \\ \mathcal {L}(C_j\cup C_k)= & p(\{\tilde{x}_i\mid i\in C_j\}\cup \{\tilde{x}_i\mid i\in C_k\}|\mathcal {H}). \end{aligned}$$

Allowing clusters to merge can lead to the MCMC being stuck in a local optimum, since it only requires a single move to merge clusters but potentially many moves to entirely reverse a merge. To mitigate this, we propose splitting clusters. Let $\textbf{H}_s = \{H_{j_1},\dots ,H_{j_{|C_j|}}\}$ be the HSACR segments in cluster $C_j$ sorted by their means, i.e., $\langle {\{\tilde{x} | \tilde{x}\in H_{j_k}\}}\rangle \le \langle {\{\tilde{x} | \tilde{x}\in H_{j_{k+1}}\}}\rangle$. We calculate the marginal likelihoods of bifurcating $\textbf{H}_s$ at every possible index *i*,

$$\begin{aligned} \mathcal {L}_i := p\left( \bigcup \nolimits _{k=1}^{i} H_{j_i}|\mathcal {H}\right) \times p\left( \bigcup \nolimits _{k=i+1}^{|C_j|} H_{j_i}|\mathcal {H}\right) , \end{aligned}$$

construct a probability distribution proportional to the marginal likelihoods, and draw from that distribution to propose a bifurcation point. We then pick a random side of the bifurcation, unassign the segments on that side, and merge these segments with another cluster according to the probabilities defined in (13). We observed that even with a well-calibrated DP prior (see Appendix 2), the influence of the prior in large ACDP clusters would often overpower smaller subclonal clusters, forcing them to merge erroneously. To mitigate this, we implemented a procedure to distinguish clonal HSACR segments from subclonal segments based on the HF described in Appendix 3, and leverage it to run the ACDP separately on each class. Similar to the ADP, we use the the ACDP as a stochastic optimizer, running it until it reaches an equilibrium and then selecting the maximum likelihood MCMC sample for the final output.

### Qualitative assessment on real tumors

We downloaded tumor BAMs aligned to hg19 for 16 Richter’s Transformed CLL samples from Klintman et al. [44]. We first realigned each of the BAMs to hg38 using bwa-mem [52] and samtools [53]. We then ran every sample through each of the five evaluated tools, using the raw data collection steps and method settings as described in Benchmarking. The segmentation file results for each algorithm can be found in Additional file 1.

### Benchmarking

We sought to rigorously benchmark HapASeg against other state-of-the-art methods across a wide range of karyotypic complexities, tumor purities, sequencing modalities, and sample types. To this end, we developed a suite of tools for simulating tumor coverage and variant profiles from arbitrary copy number profiles called CNV-Suite. The source code for this package can be found on github. Prior approaches to benchmarking CNV callers on simulated data fail to reflect the complex noise and correlation structure of real sequencing coverage [35, 42, 46–48], leading to biased results. In contrast, CNV-Suite takes variant counts and coverage profiles calculated from real diploid samples as input, and linearly scales these values based on the desired copy number profile to create simulated tumor count data with realistic noise as well as ground-truth segmentation files. Using CNV-Suite, we benchmarked CNV calling performance across five methods: HapASeg, ASCAT, Facets, HATCHet, and GATK CNV; nine tumor purities: 0.1, 0.2, $\dots$, 0.9; four sample types: fresh frozen whole genome, high quality FFPE whole genome, heavily degraded FFPE whole genome, and fresh frozen whole exome (using the TWIST bait set); and 50 simulated tumor profiles. For each of these 1800 combinations, we created simulated input data files for each of the five evaluated methods and compared the accuracy of their resulting calls to the simulation ground truth.

#### Simulating tumor samples

Each copy number calling method fundamentally requires (i) an allele counts file for each of the SNPs in the tumor of interest and (ii) a coverage file for each of its coverage windows. Typically, each method generates these files by processing sequencing reads from a Tumor-Normal pair. For our simulations, we perform the BAM processing step one time on a reference sample for each sample type, and create simulated tumor counts based on these references. By reducing compute-intensive BAM processing steps to one run per sample per method and re-using those raw data for many simulated profiles, we are able to greatly expand the number of benchmarking samples. All sequencing data used for benchmarking were aligned to the hg38 reference genome.

Each simulated tumor sample is based on sequencing data from a real, reference diploid sample. For the fresh frozen whole genome, we use the Illumina platinum sequencing of NA12878 as our reference. For the whole exome sequencing, we use a TWIST capture library of NA12878 from the Broad Genomics Platform benchmarking efforts. For the FFPE samples, we use two FFPE Richter’s Transformed CLL WGS from a previous study (CH1032:FFPE High Quality, CH1022: FFPE Degraded) [43, 44]. Sample CH1022 is not fully diploid; however, we limited the simulations to chromosomes that we identified as confidently diploid (see hatched chromosomes in 2). For all of the samples, we computed variant counts and coverage profiles for the diploid normal samples using each method’s preferred or provided BAM-processing modules, ensuring that each method used simulated data with its expected format and potential biases. Allele counts are calculated at the variant sites of NA12878. NA12878 was chosen for its complete phase information and abundance of sequencing data. The complete phasing information for each variant is required to properly simulate allele-specific copy number changes.

The simulated copy number profiles were generated by randomly sampling hyperparameters for the number of subclones (0–2), the number of arm and focal events, and proportion of clonal event for the sample. Copy number events were then randomly added to a particular allele (maternal or paternal) on the initially diploid copy profile. Focal events follow the previously reported distributions of event lengths [5]; however, we also add a number of hyper-focal events of length < 150kb in order to benchmark each method’s ability to call short segments. Each simulated profile is available in Additional file 3.

To create simulated input files for each (method, profile, sample type, purity) combination, we first find the total allele counts and coverage counts from the files generated by running the particular method’s pre-processing functions on sequencing files from the diploid sample for the corresponding sample type. Then, to simulate coverage at a given clonal locus $\mathrm{Cov}_{\ell }$ with $X_A$ maternal copies and $X_B$ paternal copies, we simply scale the diploid coverage $\mathrm{Cov}_d$ to the coverage implied by the purity *p* and the total copy state $\tau = X_A + X_B$, i.e.,

$$\begin{aligned} \mathrm{Cov}_{\ell } = \mathrm{Cov}_d(1 - p) + p \cdot \frac{\tau \cdot \mathrm{Cov}_d }{2} \end{aligned}$$

For loci with subclones at subclonal cancer cell fractions $f_1, f_2, \dots , f_i$, the coverage is scaled by the weighted allelic copies of each of the clones $X^{f_i}_A , X^{f_i}_B$ and the clonal copies $X^c_A + X^c_B$, where $\tau ^z = X^z_A + X^z_B$ is the total copy number of clone *z*.

14 $$\begin{aligned} \mathrm{Cov}_{\ell } = \mathrm{Cov}_d\left[ (1 - p) + \frac{p[\tau ^c(1 - \sum _i f_i) + \sum _i f_i \tau ^i]}{2} \right] \end{aligned}$$

Most methods expect measured coverage to take integer values, however, HapASeg uses fragment-length normalized coverage $\mathrm{Cov}_d$, which can take any positive real number value. To avoid imposing generative modeling assumptions on our coverage simulations, we pass $\lfloor \mathrm{Cov}_d\rfloor$ to downstream estimation methods.

The simulated sequencing read counts for each allele $A_{\ell }, B_{\ell }$ at locus $\ell$ are computed by first calculating the simulated total allele counts $T_{\ell }$ at the locus by taking the floor of the scaled diploid read counts as in (14). The allele-specific read counts are then drawn from a binomial as

$$\begin{aligned} A_{\ell } \sim \text {B}(T_{\ell }, r_{\ell }), B_{\ell } = T_{\ell } - A_{\ell } \end{aligned}$$

where

$$\begin{aligned} r_{\ell } = \frac{(1-p) + p\left[ X^c_A(1 - \sum _i f_i) + \sum _i f_i X^{f_i}_A \right] }{2(1-p) + p \left[ \tau ^c (1 - \sum _i f_i) + \sum _i f_i \tau ^{f_i} \right] } \end{aligned}$$

#### TCGA matched trio comparison

A small subset of the cancer cases studied as part of TCGA had samples of the same tumor biopsy preserved in separate fresh frozen and FFPE aliquots. We curated a set of nine such cases with whole genome sequencing from four tumor types (3 LUAD, 2 COAD, 1 BRCA, 3 UCEC). The sample metadata is provided in Additional file 5. We ran each fresh frozen and FFPE sample through HapASeg and the four additional methods to estimate sCNAs. To measure the accuracy of the models on the FFPE samples, we computed AAD scores as described in Comparing results using the fresh frozen HapASeg results as ground truth and normalized the mean coverage levels of each sample to make the results comparable. The AAD scores for the matched TCGA comparisons are in general higher than in the simulated benchmarking. This may be partially attributed to the fact that the fresh frozen and FFPE samples are comprised of different tumor cells, and may have different purity or clonal compositions despite being derived from the same biopsy.

#### Normal samples

Some methods, such as HATCHet, require allele counts and coverage from a normal sample in order to run, while each of the other methods can optionally take a normal sample as input. In an attempt to benchmark each method on equal footing, we decided to let every method use a matched normal sample. For the matched samples, we located sequencing data from alternative libraries of the same tissue, when possible. For the fresh frozen WGS, we used a BAM from a separate library of NA12878 made available in a recent publication [54], and for the fresh frozen exome, we used a separate TWIST sequencing of NA12878. Clinical FFPE samples generally do not have paired FFPE normal tissue and instead use fresh frozen blood normals, which renders the normal coverage less useful to regress out coverage noise. To reflect the difficulty of copy number calling on FFPE clinical samples in our benchmarking study, we used matched fresh frozen blood normals for each of the FFPE clinical samples. To generate allele counts and coverage for each matched normal sample, we ran the same preprocessing steps as for the simulated tumor samples.

#### Panel of normals

GATK CNV can optionally use a panel of normals (PoN) in place of a matched normal to regress out coverage artifacts. We created a WGS PoN using 50 random samples from the 1000 genomes (1kG) stage 3 PCR-free whole genome cohort [49], and a WES PoN using six TWIST WES samples sequenced at the Broad Genomics Platform. FFPE samples were given the 1kG WGS PoN since, to the best of our knowledge, no WGS FFPE PoN is available. We did not see a noticeable performance improvement for GATK CNV between PoN normalization and normal sample normalization. Unless otherwise specified, all GATK CNV analyses were performed using the corresponding PoN.

#### Running competing tools

We ran each tool using its default configuration. Details of the preprocessing steps, run commands, and options used for each method can be found in Appendix 4. We ran all benchmarking using an in-house workflow management system.

#### Comparing results

We compared the copy number profiles from each of the evaluated methods across the range of simulated purities. Only a subset of the methods in our benchmarking set return absolute copy number estimates; however, all methods besides Facets compute allelic coverage estimates. We therefore chose to evaluate methods by comparing their allelic coverage estimates to the simulated ground truth profile. In particular, we developed the average absolute difference (AAD) score to evaluate the quality of coverage estimates given a ground truth coverage profile (see Additional file 2: Fig. S2 for illustration). The AAD score is defined as the length-weighted absolute difference in allelic coverage across the autosomes between the estimated and ground truth samples. In particular, let the estimated allelic coverage segments for a particular simulated copy profile *e* at purity *p* and method *m* be denoted as $S_{(e, p, m)} = \{s_1, \dots , s_i, \dots \}$ and the ground truth segments as $G_{(e, p)} = \{s_1, \dots , s_j, \dots \}$ where $s_k = (\text {start}_k, \text {end}_k, \mu ^{\text {major}}_k, \mu ^{\text {minor}}_k)$ and $\mu _k$ is the allelic coverage for segment *k*. We can compute the intersection of these segment intervals $I_{(e,p,m)} = \{t_1, \dots , t_{\ell }, \dots \}, t_{\ell } = (\text {start}_{\ell }, \text {end}_{\ell }, \mu ^{\text {major}}_{S, \ell }, \mu ^{\text {minor}}_{S, \ell }, \mu ^{\text {major}}_{G, \ell }, \mu ^{\text {minor}}_{G, \ell })$ and compute the AAD score as

$$\begin{aligned} \mathrm{AAD}^{\text {naive}}_{(e,p,m)} = \frac{\sum _{\ell }{\left( \text {end}_{\ell } - \text {start}_{\ell }\right) \left[ \left| \mu ^{\text {major}}_{S, \ell } - \mu ^{\text {major}}_{G, \ell }\right| + \left| \mu ^{\text {minor}}_{S, \ell } - \mu ^{\text {minor}}_{S, \ell } \right| \right] }}{\sum _{\ell }{\left( \text {end}_{\ell } - \text {start}_{\ell }\right) }} \end{aligned}$$

This comparison, however, is complicated by the fact that the evaluated methods use varying units of coverage, meaning that the ground truth coverage profiles at different purities will have varying levels of average coverage, and thus also varying AAD scales. For example, a duplicated region erroneously called as diploid will have a much higher AAD score in a sample with a purity of 0.9 than a sample with a purity of 0.1. To normalize comparisons across methods and purities, we find the optimal affine transformation $f(x) = ax + c$, which minimizes the AAD score between the intersection of a given output segmentation file $S_{(e, p, m)}$ and ground truth segments at 0.7 purity $G_{(e, 0.7)}$ for the same profile *e*. (We chose 0.7 since this is a realistic high purity tumor sample and avoids numerical instabilities.) That is,

$$\begin{aligned} \mathrm{AAD}_{(e,p,m)} = \, \underset{f}{\mathrm{min}} \, \frac{\sum _{\ell }{\left( \text {end}_{\ell } - \text {start}_{\ell }\right) \left[ \left| f(\mu ^{\text {major}}_{S, \ell }) - \mu ^{\text {major}}_{G, \ell }\right| + \left| f(\mu ^{\text {minor}}_{S, \ell }) - \mu ^{\text {minor}}_{S, \ell } \right| \right] }}{\sum _{\ell }{\left( \text {end}_{\ell } - \text {start}_{\ell }\right) }} \end{aligned}$$

We find the optimal affine transformation *f* via Powell’s method. For the heatmap result plots, we use the optimal AAD scores described here; however, when stratifying the results based on segment length, we used the ground truth segment length, rather than the length of the intersected segment.

## Supplementary information

Additional file 1. Richter’s results plots. Tables 1-16: each sheet labeled “CHXXXX” in this excel workbook contains the sCNA estimation results for sample “CHXXXX” using each of the five methods tested.

Additional file 2. Supplementary figures. Fig. S1.: HapASeg benchmarking pabel schematic. Fig. S2: AAD calculation schematic. Fig. S3: Fresh frozen WES benchmarking results heatmap. Fig. S4: TCGA matched fresh frozenand FFPE WGS results. Fig. S5: Simulated tumor karyotype summary statistics.

Additional file 3. Simulated Karyotype plots. Tables 1-50: each sheet labeled “profile_X” in this excel workbook contains contains a illustration of simulated karyotype profile X generated for benchmarking.

Additional file 4. Quantitative benchmarking results table. This workbook includes the raw segmentation file results for the quantitative benchmarking study for all tested methods and all 50 simulated tumors at a simulated purity of 0.7. Each sheet of the table contains the simulated karyotype, phylogeny of each tumor, and figures illustrating the segment level absolute errors for the estimates from each method.

Additional file 5. TCGA cohort metadata table. This table contains TCGA sample information for the FFPE trio cohort.

## Figures and Tables

**Fig. 5:**
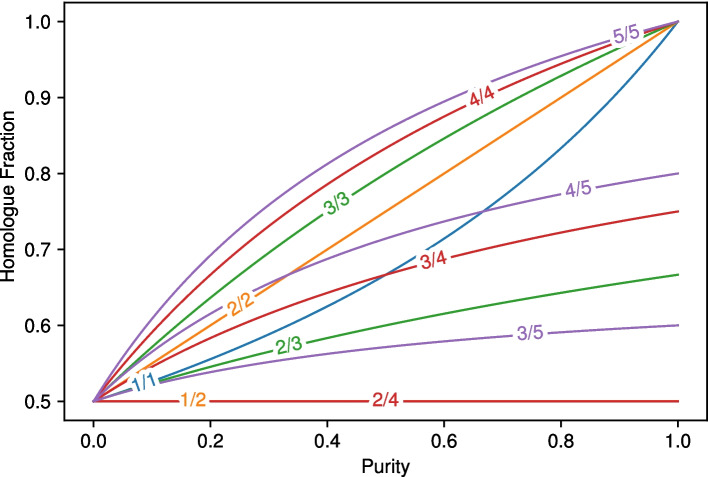
Homolog fraction as a function of tumor purity for different clonal major allele counts and tumor total copy numbers (tTCN), for tTCN values from 1 to 5. Each line is labeled as (major allele count)/(tTCN), e.g., 1/2 corresponds to a heterozygous normal diploid regions; and 2/2 corresponds to a diploid region with copy-neutral LoH. Note that the curves rarely intersect, indicating that tTCN is nearly a well-defined function of homolog fraction. However, note that this function is not perfectly well-defined: multiple homolog fractions can map to the same tTCN (where lines intersect), especially when subclonal sCNAs exist (not shown)

## Data Availability

Richter’s Transformed CLL samples along with matched blood normals were obtained from Parry et al. [43] and realigned to hg38. Original data are available at dbGaP (https://www.ncbi.nlm.nih.gov/gap/) using accession number phs002458.v2.p1 [58]. As stated in Parry et al. [43], patient samples were collected following written informed consent under IRB-approved study protocols and in compliance with all ethical regulations. WGS sequencing data for NA12878 used for benchmarking are available at the NCI Sequence Read Archive (SRA) under accession number SRX3666165 [54, 59] and from the Illumina platinum truthset, available at the European Nucleotide Archive (ENA) under accession code ERR194147 [60, 61]. WES TWIST data for NA12878 were generated by the Broad Institute Genomics Platform and are publicly available in a Terra workspace named “Broad-HapASeg-exome-validation” [62]. The matched normal, fresh frozen tumor and FFPE tumor trios from TCGA were curated from the GDC Data Portal and using dbGAP accession phs000178.v11.p8 [63]. The sample information for each trio is provided in Additional file 5. The simulated karyotype profiles, along with the associated simulated tumor data and method results can be downloaded from https://github.com/getzlab/HapASeg_manuscript.

## Appendix

### 1 Total copy number as function of homolog fraction

Here, we demonstrate that homolog fraction (HF) is a nearly well-defined function of total copy ratio. Homolog fraction *f* of a heterozygous germline SNP is a function of its allele-specific copy number on one of the two homologs $n_A$, the total copy number $\tau$, and the fraction of tumor cells $\alpha$.

15 $$\begin{aligned} f(n_A,\tau ,\alpha )= & \frac{n_A\alpha + 1(1-\alpha )}{\tau \alpha + 2(1-\alpha )}\\= & \frac{1 + \alpha (n_A - 1)}{2 + \alpha (\tau - 2)} \end{aligned}$$

The numerator is the total number of copies containing the allele; the fraction $1-\alpha$ of normal cells are diploid and thus always have one copy of the allele, while the fraction $\alpha$ of tumor cells have $n_A$ copies of the allele. The denominator is the total number of copies; diploid normal cells all have two copies, while tumor cells all have $\tau$ copies.

We plot *f* in Fig. 5 for all purities at a variety of allele-specific copy numbers. We see that, with a few exceptions (detailed below), the curves never intersect, demonstrating that total copy number is nearly a well-defined function of HF and purity, and thus ideal to use as a strong prior for total copy segmentation.

For all allele-specific copy numbers with $\tau \le 5$, the exceptions are:

1. At any purity, a balanced diploid ($n_A = 1$, $\tau = 2$) region has the same homolog fraction ($\frac{1}{2}$) as a balanced tetraploid (genome doubled) region ($n_A = 2$/$\tau = 4$), but will have differing total copy ratios respectively corresponding to total ploidies of 2 and 4. Thus, a 1/2 region immediately adjacent to a 2/4 region would not be distinguishable from homolog fraction segmentation alone. Indeed, this will be the case for any balanced region.
2. At purity = 0.5, a haploid (1/1) region has the same homolog fraction ($\frac{2}{3}$) as a tetraploid (3/4) region.
3. At purity = 1/3, a region of copy-neutral loss-of-heterozygosity (2/2) has the same homolog fraction ($\frac{2}{3}$) as a pentaploid (4/5) region.
4. At purity = 2/3, a haploid region and a pentaploid region (4:1) both have homolog fraction 0.7
5. At purity = 100%, all loss-of-heterozygosity regions have the same homolog fraction (1).

Furthermore, ambiguities only arise if degenerate HF/TCR pairings occur in immediately adjacent segments (e.g., a balanced diploid segment next to a balanced tetraploid segment).

Note that more ambiguities can arise if subclones are present. Let the fraction of the *i*th subclone be $s_i$; $\textbf{s}= \{s_1,\dots ,s_N\}$, $\sum _{i=1}^N s_i = 1$. Then $n_A$ and $\tau$ in (15) become a weighted average of allelic copies over subclones.

$$\begin{aligned} n'_A(\textbf{s})= & \sum \limits _{i=1}^N s_i n_{A_i}\qquad \tau '(\textbf{s}) = \sum \limits _{i=1}^N s_i \tau _i\\ f(n'_A(\textbf{s}),\tau '(\textbf{s}),\alpha )= & \frac{1 + \alpha (n'_A(\textbf{s}) - 1)}{2 + \alpha (\tau '(\textbf{s}) - 2)} \end{aligned}$$

(In a region with no subclones, $s_1 = 1$; $\sum _{i=2}^N s_i = 0$, recovering (15).)

### 2 Dirichlet Process details

A Dirichlet Process (DP) is a commonly used prior for mixture models with variable numbers of components. It is a prior on the number of datapoints in each component. The DP prior can be derived by starting with a mixture model with a fixed number of components (*K*), with a symmetric Dirichlet-categorical prior on the assignment probabilities of each datapoint.

$$\begin{aligned} x_i|\{\theta _k\}_{k=1}^K,c_i\sim & p(\theta _{c_i})\\ c_i|\{p_k\}_{k=1}^K\sim & \mathrm{Cat}(p_1,\dots ,p_K)\\ \{p_k\}\sim & \mathrm{SymDir}(\alpha /K). \end{aligned}$$

We typically use a Gibbs sampler when sampling this DP mixture model via MCMC; we take one datapoint, remove it from the cluster it is currently assigned to, and compute the probability of assigning it to all other clusters, conditioned on the assignments of every other datapoint. This probability (that the *i*th datapoint is assigned to component $\tilde{c}$, given the assignments of all other data points) is

$$\begin{aligned} p(c_i=\tilde{c}|\underbrace{c_1,\dots ,c_{i-1},c_{i+1},\dots , c_N}_{\textbf{c}_{-i}}) = \frac{p(\overbrace{c_1,\dots ,c_i=\tilde{c}, \dots ,c_N}^{\textbf{c}})}{p(\textbf{c}_{-i})}, \end{aligned}$$

by definition of conditional probability. Without loss of generality, let $i=1$ and $\tilde{c} = 1$, since the ordering of data points and clusters is arbitrary. The numerator is the Dirichlet-Categorical likelihood, after marginalizing out $\textbf{p}$:

$$\begin{aligned} p(\textbf{c})= & \int d\textbf{p}\, \mathrm{Cat}(c_1 = 1|{\textbf {p}})\left[ \prod _{i=2}^N \mathrm{Cat}(c_i|\textbf{p})\right] \mathrm{SymDir}(\textbf{p}|\alpha /K)\\ & \propto \int d\textbf{p}\, p_1^{1 + n_1 + \alpha /K - 1}\prod _{k=2}^{K} p_k^{n_k+\alpha /K - 1}. \end{aligned}$$

where $n_1$ is the number of points already in cluster 1, i.e., not counting $x_1$. This integral has closed form—it’s the multivariate beta function:

16 $$\begin{aligned} \int d\textbf{p}\, p_1^{1 + n_1 + \alpha /K - 1} \prod _{k=2}^K p_k^{n_k+\alpha /K - 1}= & \frac{\Gamma (1 + n_1 + \alpha /K)\prod _{k=2}^K \Gamma (n_k + \alpha /K)}{\Gamma (1 + \sum _{k=1}^K n_k + \alpha /K)}\\= & \frac{\Gamma (1 + n_1 + \alpha /K)\prod _{k=2}^K \Gamma (n_k + \alpha /K)}{\Gamma (N + \alpha )}. \end{aligned}$$

The likelihood in the denominator is similar:

17 $$\begin{aligned} p(\textbf{c}_{-i})= & \int d\textbf{p}\left[ \prod _{i = 2}^N \mathrm{Cat}(c_i|\textbf{p})\right] \mathrm{SymDir}(\textbf{p}|\alpha /K)\\ & \propto \int d\textbf{p}\, p_1^{n_1 + \alpha /K - 1} \prod _{k=2}^K p_k^{n_k+\alpha /K - 1}\\= & \frac{\prod _{k=1}^K \Gamma (n_k + \alpha /K)}{\Gamma (N + \alpha - 1)}. \end{aligned}$$

Note the $-1$ term in the denominator, since we have one fewer datapoint.

Putting (16) and (17) together, we have

18 $$\begin{aligned} \frac{p(\textbf{c})}{p(\textbf{c}_{-i})} = \frac{\Gamma (1 + n_1 + \alpha /K)\Gamma (N + \alpha - 1)}{\Gamma (n_1 + \alpha /K)\Gamma (N + \alpha )}=\frac{n_1 + \alpha /K}{N + \alpha - 1} = \frac{n_{\tilde{c}} + \alpha /K}{N + \alpha - 1}. \end{aligned}$$

where the last equality arises from the fact that we took $\tilde{c} = 1$ without loss of generality. If the number of clusters is variable, we take the limit $K\rightarrow \infty$, yielding

$$\begin{aligned} \lim _{K\rightarrow \infty } p(c_i=\tilde{c}|c_1,\dots ,c_{i-1},c_{i+1},\dots , c_N) = \frac{n_{\tilde{c}}}{N + \alpha - 1} \end{aligned}$$

The probability of opening a new cluster is

$$\begin{aligned} p_\text {new}:= p(c_i \ne c_k\forall k\le \kappa |\textbf{c}_{-i}) \end{aligned}$$

where $\kappa$ is the current number of clusters. Note that

$$\begin{aligned} p(c_i \ne c_k\forall k\le \kappa |\textbf{c}_{-i}) = p(c_i = c_k\forall k> \kappa |\textbf{c}_{-i}), \end{aligned}$$

and that $n_k=0\;\forall \, k>\kappa$. Plugging into (18), the probability of opening a new cluster becomes

19 $$\begin{aligned} p_\text {new} =p(c_i = c_k\forall k> \kappa |\textbf{c}_{-i})&=\lim _{K\rightarrow \infty } \sum \limits _{k = \kappa + 1}^K \frac{\alpha /K}{N + \alpha - 1}\\&=\lim _{K\rightarrow \infty }(K - (\kappa + 1))\frac{\alpha /K}{N + \alpha - 1}\\&=\frac{\alpha }{N + \alpha - 1}. \end{aligned}$$

What if we are moving more than one datapoint at once, as is the case when we combine segments containing multiple SNPs In that case, we have

$$\begin{aligned} p(\underbrace{\{c_{z_i}\}_{i=1}^M}_{\textbf{c}_\text {move}}=\tilde{c} |\underbrace{\textbf{c}\setminus \textbf{c}_\text {move}}_{\textbf{c}_{-\text {move}}}) = \frac{p(\overbrace{c_1,\dots ,c_{z_1}=\tilde{c},c_{z_2}=\tilde{c},\dots ,c_{z_M}=\tilde{c},\dots ,c_N}^{\textbf{c}})}{p(\textbf{c}_{-\text {move}})}. \end{aligned}$$

The math proceeds similarly as when moving a single datapoint. Again, WLOG, let $z_1,\dots ,z_M=1,\dots ,M$ and $\tilde{c} = 1$. Then

20 $$\begin{aligned} p(\textbf{c})= & \int d\textbf{p}\left[ \prod _{i=1}^M \mathrm{Cat}(c_i = 1|{\textbf {p}})\right] \left[ \prod _{i={M+1}}^N \mathrm{Cat}(c_i|\textbf{p})\right] \mathrm{SymDir}(\textbf{p}|\alpha /K)\\ & \propto \int d\textbf{p}\, p_1^{M + n_1 + \alpha /K - 1} \prod _{k=2}^K p_k^{n_k + \alpha /K - 1}\\= & \frac{\Gamma (M + n_1 + \alpha /K)\prod _{k=2}^K \Gamma (n_k + \alpha /K)}{\Gamma (N + \alpha )} \end{aligned}$$

and

21 $$\begin{aligned} p(\textbf{c}_{-\text {move}})= & \int d\textbf{p}\left[ \prod _{i=M+1}^N \mathrm{Cat}(c_i|\textbf{p})\right] \mathrm{SymDir}(\textbf{p}|\alpha /K)\\\propto & \int d\textbf{p}\, p_1^{n_1 + \alpha /K - 1} \prod _{k=2}^K p_k^{n_k + \alpha /K - 1}\\= & \frac{\prod _{k=1}^K \Gamma (n_k + \alpha /K)}{\Gamma (N + \alpha - M)}. \end{aligned}$$

The ratio of (19) and (20) is

22 $$\begin{aligned} \frac{p(\textbf{c})}{p(\textbf{c}_{-\text {move}})} = \frac{\Gamma (M + n_1 + \alpha /K)\Gamma (N + \alpha - M)}{\Gamma (n_1 + \alpha /K)\Gamma (N + \alpha )}. \end{aligned}$$

To further simplify this, recall that

$$\begin{aligned} \frac{\Gamma (x - y)}{\Gamma (x)} = \frac{1}{\prod _{i=1}^y(x - i)}\qquad \frac{\Gamma (x + y)}{\Gamma (x)} = \prod _{i=0}^{y-1}(x + i)\qquad x\in \mathbb {R}^+, y\in \mathbb {Z}^+, \end{aligned}$$

so (21) becomes

$$\begin{aligned} p(\textbf{c}_\text {move}|\textbf{c}_{-\text {move}})= & \lim _{K\rightarrow \infty }\frac{\Gamma (M + n_{\tilde{c}} + \alpha /K)\Gamma (N + \alpha - M)}{\Gamma (n_{\tilde{c}} + \alpha /K)\Gamma (N + \alpha )}\\= & \frac{\prod _{i=0}^{M - 1}(n_{\tilde{c}} + i)}{\prod _{i=1}^{M}(N + \alpha - i)}. \end{aligned}$$

In practice, it is computationally simpler to use the expression in (21) (after taking the limit $K\rightarrow \infty$), since numerical computing packages provide efficient functions for evaluating $\log \Gamma (x)$ directly. The probability of opening a new cluster is thus

$$\begin{aligned} p(\{c_{z_i}\}_{i=1}^M = c_k\forall k>\kappa |\textbf{c}_{-\text {move}})= & \lim _{K\rightarrow \infty }\sum \limits _{k=\kappa + 1}^K \frac{\Gamma (M + \alpha /K)\Gamma (N + \alpha - M)}{\Gamma (\alpha /K)\Gamma (N + \alpha )}\\= & \frac{\Gamma (N + \alpha - M)}{\Gamma (N + \alpha )}\lim _{K\rightarrow \infty }\sum \limits _{k=\kappa + 1}^K \frac{\Gamma (M + \alpha /K)}{\Gamma (\alpha /K)}\\= & \frac{\Gamma (N + \alpha - M)}{\Gamma (N + \alpha )}\lim _{K\rightarrow \infty }(K-(\kappa +1))\prod _{i=0}^{M-1}(\alpha /K + i)\\= & \frac{\Gamma (N + \alpha - M)}{\Gamma (N + \alpha )}\alpha \Gamma (M). \end{aligned}$$

Unlike in the case of moving a single datapoint at a time, we cannot use the probabilities $p(\textbf{c}_\text {move}|\textbf{c}_{-\text {move}})$ as-is. The event space of $p(\textbf{c}_\text {move})$ comprises all possible assignments of each datapoint in $\textbf{c}_\text {move}$, with probabilities normalized with respect to this event space. For example, suppose we are moving *M* datapoints $c_{z_1},c_{z_2},\dots ,c_{z_M}$. The event space is the Cartesian product $\lim _{K\rightarrow \infty }[\{c_{z_1}=k\}_{k=1}^K\times \{c_{z_2}=k\}_{k=1}^K\times \dots \times \{c_{z_M}=k\}_{k=1}^K]$. However, we are only interested in the event space for which *k* is constant across all datapoints, i.e., $\lim _{K\rightarrow \infty }\{c_{z_1}=k,c_{z_2}=k,\dots ,c_{z_M}=k\}_{k=1}^K$. We must re-normalize our probabilities, like so:

$$\begin{aligned} p'(\{c_{z_i}\}_{i=1}^M = \tilde{c}|\textbf{c}_{-\text {move}})\equiv & p(\{c_{z_i}\}_{i=1}^M = \tilde{c}|\textbf{c}_{-\text {move}},c_{z_1}=c_{z_2}=\cdots =c_{z_M})\\= & \frac{p(\{c_{z_i}\}_{i=1}^M=\tilde{c}|\textbf{c}_{-\text {move}})}{\sum _{k=1}^\kappa p(\{c_{z_i}\}_{i=1}^M=c_k|\textbf{c}_{-\text {move}}) + p_\text {new}}. \end{aligned}$$

### 3 ACDP Implementation Details

#### Distinguishing clonal vs. subclonal events

Most sCNAs are clonal, so most HSACR segments will be assigned to a handful of ACDP clusters corresponding to the clonal states of $0,1,2,\dots$ copies of each allele. The Dirichlet process prior favors a uniform distribution of cluster sizes and penalizes small clusters; this uniform distribution often forces small clusters, which correspond to subclonal copy states, to merge with nearby clonal clusters, resulting in a loss of subclonal calling accuracy. To mitigate this, we separate clonal and subclonal HSACR segments and run the ACDP clustering on each of them separately. We separate clonal from subclonal segments using a two-step process. The first step relies solely on HF, which provides an extremely clean signal but does not unambiguously indicate HSACR. The second step looks at HSACR, which is noisier than HF but can distinguish between copy number states (Fig. 5).

#### HF step

A clonal segment has homolog fraction

$$\begin{aligned} \alpha (p, n_A, \tau ) = \frac{1 +p (n_A - 1)}{2 (1 - p) + p \tau } \end{aligned}$$

where $p\in [0, 1]$ is the purity of the sample, $\tau \in \mathbb {Z}^+$ is the clonal ploidy of the segment, and $n_A \in \{0,\dots ,\tau \}$ is the clonal copy number of homolog A for the segment. The likelihood of the *i*th ACR segment $\Xi _i$ is

$$\begin{aligned} \mathcal {L}_i(p,\tau ,n_A|A_i, B_i) = \mathrm{Beta}(\alpha (p, n_A, \tau ); A_i + 1, B_i + 1) \end{aligned}$$

where $A_i$ and $B_i$ are the read counts assigned to the A and B homologs within the $\Xi _i$. For a given purity $p^*$, we maximize for each segment

$$\begin{aligned} \hat{\tau }_i, \hat{n}_{a,i} = \mathrm {\underset{\tau ,n_A}{\arg \max }}\mathcal {L}_i(\tau ,n_A|A_i, B_i, p^*) \end{aligned}$$

over all $\tau \in \{0\dots 7\}$, $n_A\in \{0\dots 3\}$. We then maximize the overall likelihood

$$\begin{aligned} \mathcal {L}(p^*|\{A\},\{B\}) = \prod _i \mathcal {L}_i(\hat{\tau }_i, \hat{n}_{a,i}|A_i, B_i, p^*) \end{aligned}$$

over all purity values $p^*\in [0,1]$ with step size $10^{-4}$, yielding optimal purity

$$\begin{aligned} \hat{p}^*= \mathrm {\underset{p^*}{\arg \max }}\mathcal {L}(p^*|\{A\},\{B\}) \end{aligned}$$

Given $\hat{p}^*$, we sort the ACR segments by their genomic mass (length times ploidy) $\Xi _{s_1},\dots ,\Xi _{s_N}$ and compute the cumulative log-likelihood

$$\begin{aligned} \mathcal {L}_\text {cum}(k) = \sum \limits _{i=1}^k\log \mathcal {L}_{s_i}(\hat{p}^*, \hat{n}_{a,s_i}, \hat{\tau }_{s_i}|A_{s_i}, B_{s_i})\qquad k\le s_N \end{aligned}$$

and find the elbow point $\iota (k)$ of this curve as a threshold to identify segments that are confidently clonal. These segments undergo a full run of the ACDP, generating clusters $\textbf{C}^*= \{C^*_1\dots C^*_{|\textbf{C}^*|}\}$. We assume that each cluster corresponds to a distinct clonal allelic copy number state. These will serve as a seed to identify additional clonal segments based on their full ACR values.

#### HSACR step

Recall that HSACR has units of sequencing coverage, since an HSACR segment $\xi$ is the product of the segment’s HF (which is a dimensionless scale factor) and its TCR (which HapASeg measures in fragment length-normalized coverage). We must associate allelic coverage levels with discrete clonal allelic copy number states.

To do so, we begin by taking all clusters $\textbf{C}^*$ from the previous step and computing a gaussian likelihood for each cluster $C^*_k$ using the associated purity $\hat{p}^*$, allelic copy number $n_k$, and coverage scale factor $\phi$, such that

$$\begin{aligned} \mathcal {L}_k(n_k,\phi |\{\tilde{x}\mid i\in C^*_k\},\hat{p}^*,\sigma _k) = \prod _{i\in C^*_k}\mathcal {N}(\tilde{x}_i;\mu (n_k,\phi ,\hat{p}^*),\sigma _k), \end{aligned}$$

$$\begin{aligned} \mu (n_k,\phi ,\hat{p}^*) = \phi (1 - \hat{p}^*)+n_k\hat{p}^*. \end{aligned}$$

Note that $\sigma _k$ is simply the cluster’s empirical standard deviation $\sqrt{\mathrm{Var}(\{\tilde{x}_i | i\in C^*_k\})}$, and is not a free parameter.

We find the optimal scale factor $\hat{\phi }$ by performing a grid search similar to the one described above for $\hat{p}^*$. We optimize $\phi$ as across all clusters in $\textbf{C}^*$ as

$$\begin{aligned} \hat{\phi }= \mathrm {\underset{\phi }{\arg \max }}\prod _{k=1}^{|\textbf{C}^*|} \mathcal {L}_k(\phi |\{\tilde{x}_i \mid i\in C^*_k\},\hat{p}^*,\hat{n}_k, \sigma _k) \end{aligned}$$

where for each $\phi$ we compute the assignment for each $C^*_k$ to a clonal copy state $n_k\in \{0\dots 5\}$ as

$$\begin{aligned} \hat{n}_k = \mathrm {\underset{n_k}{\arg \max }}\mathcal {L}_k(n_k|\{\tilde{x}\mid i\in C^*_k\},\hat{p}^*,\sigma _k,\phi ). \end{aligned}$$

We then assess the clonality of clusters $C_{\ell }$ in $\xi _{clonal}$ by comparing the Gaussian likelihood of data points

$$\begin{aligned} p(C_{\ell }|\mu ^{m^*}_{\eta ^*, p^*}, \sigma _{\ell }) = \sum \limits _{x_i \in \mathcal {R}(C_{\ell })} \log \mathcal {N}(x_i; \mu ^{m^*}_{\eta , p^*}, \sigma _{\ell }) \end{aligned}$$

to a null model

23 $$\begin{aligned} p_{0}(C_{\ell }) = \sum \limits _{x_i \in \mathcal {R}(C_{\ell })} \log U(x_i; \mathrm{min}(\mathcal {R}(C_{\ell })), \mathrm{max}(\mathcal {R}(C_{\ell })) , \end{aligned}$$

where $m^*$ is the most likely allelic copy level for cluster $\ell$. The null distribution is a uniform distribution with the same domain as the data points in $D_{C_{\ell }}$. Clusters where $p(C_{\ell }|\mu ^{m^*}_{\eta ^*, p^*}, \sigma _{\ell })> p_{0}(C_{\ell })$ and which comprise greater than 1% of the total coverage windows are labeled as confidently clonal, denoted $\mathfrak {C}$. We then construct our final set of clonal $\mathfrak {S}_{clonal}$ and sub-clonal segments $\mathfrak {S}_{subclonal}$ by initially setting $\mathfrak {S}_{clonal} = \mathcal {S}(\mathfrak {C})$ and $\mathfrak {S}_{subclonal} = \mathcal {S}(\xi _{clonal} \setminus \mathfrak {C})$. We also wish to re-assess the segments that were originally deemed non-clonal by testing whether they match the distribution of an existing clonal segment better than a null distribution. That is, we assign segments $S_j, S_j \notin \mathcal {S}(\xi _{clonal})$ to $\mathfrak {S}_{clonal}$ or $\mathfrak {S}_{subclonal}$ as

$$\begin{aligned} S_j \rightarrow \mathfrak {S}_{clonal} \quad \text {iff} \quad p(S_j)> p_0(S_j), \end{aligned}$$

with

$$\begin{aligned} p(S_j)= & \underset{C_w}{\mathrm{max}}\sum \limits _{x_i \in S_j} \log \mathcal {N}(x_i; \overline{\mathcal {R}(C_w)}, \sqrt{\mathrm{Var}(\mathcal {R}(C_w)}), C_w \in \mathfrak {C}\\ p_0(S_j)= & \sum \limits _{x_i \in \mathcal {R}(S_j)} \log U(x_i; \mathrm{min}(\mathcal {R}(S_j), \mathrm{max}(\mathcal {R}(S_j)). \end{aligned}$$

With the assignments for $\mathfrak {S}_{clonal}$ and $\mathfrak {S}_{subclonal}$ in hand, we finally run ACDP clustering on each segment set independently, and combine the resulting clusters from the draw from each chain after burn-in to produce our final results.

### 4 Running sCNA methods

#### ASCAT

We installed ASCAT v3.1.1 from the github repository in a docker container with its dependencies [32]. ASCAT only uses allele count data to perform copy number calling, and provides its own basic allele counting method. We tested the difference between allele counts at NA12878 variant sites from MuTect and ASCAT prepare.HTS and found very strong agreement. We chose to move forward with the MuTect counts since these counts were already generated for use by HapASeg. Following our protocol for HapASeg, we removed sites with greater than 5% MAPQ0 reads, or with greater than 10% of reads filtered by MuTect standard filters, to remove likely sequencing artifacts. We then filtered on the ASCAT’s WGS loci list as recommended in their documentation. The matched normal allele counts were computed using MuTect and merged with the passing “tumor” sites. Allele counts for the simulations were generated using the procedure described above in Simulating tumor samples section, and the resulting counts were converted to LogR and AAF format for input to ASCAT. We ran ASCAT as using the default settings. This included correcting the LogR file using the GC and RT correction files provided in their repository. The raw allelic coverage estimates for downstream analysis were taken from the segments_raw.txt ASCAT output file.

#### Facets

We installed Facets v0.6.2 from the available github repository in a docker container with its dependencies [41]. Facets only uses allele count data to perform copy number calling, and provides its own basic allele counting method. We tested the difference between allele counts at NA12878 variant sites from MuTect and Facets SNP Pileup and found very strong agreement. We chose to move forward with the MuTect counts since these counts were already generated for use by HapASeg. Following our protocol for HapASeg, we removed sites with greater than 5% MAPQ0 reads, or with greater than 10% of filtered reads by MuTect standard filters, to remove likely sequencing artifacts. We then filtered using the Facets WGS loci list from dbSNP as recommended in their documentation. The matched normal allele counts were also computed using MuTect and were merged with the passing “tumor” sites. Allele counts for the simulations were generated using the procedure described above in Simulating tumor samples section, and the resulting counts were converted to the SNP counts format for Facets. We ran Facets as recommended in the documentation with default settings of minimum SNP depth of 10 and critical value of 150.

Facets does not return, or in fact even compute, raw allele-specific coverage as part of its algorithm. Instead, it returns estimated allelic copy number that it computes after fitting a comb. We therefore use these smoothed estimates, which can be found in the facets segmentation file generated by the emcncf function, for downstream comparison.

#### GATK CNV

For our GATK CNV benchmarking analyses, we followed the procedure on the GATK CNV tutorial. We used the publicly available broadinstitute/gatk:4.0.1.1 GATK4 docker container, which was also used in the tutorials, for all of our GATK CNV analyses [34]. We used the standard public hg38 WGS calling intervals file with zero padding for our WGS preprocessing and the TWIST target intervals with a 250bp padding for WES. We excluded the sex chromosomes for all analyses since their analysis would require generating sex chromosome–specific PoNs. Allele counts were computed using CollectAllelicCounts on the set of NA12878 variants, and coverage was computed with CollectFragmentCounts using the recommended interval size of 1kb. Simulated tumor allele counts and coverage were generated with the procedure described above in Simulating tumor samples section, and underwent PoN and GC correction using the DenoiseReadCounts function. These denoised counts were then used as input for copy number calling by the ModelSegments function, which was run with default options. Downstream results were calculated from the modelFinal.seg segmentation file.

To generate the PoN files for denoising, we first ran CollectFragmentCounts on each of the panel BAMs using the same WGS/WES specific interval list as described above in Panel of normals section. The fragment counts were then aggregated into a PoN using the CreateReadCountPanelOfNormals function with the minimum interval median percentile option set to 5, and all other options set to default. The PoN files are available in the benchmarking data bucket.

#### HapASeg

Allele counts were generated using MuTect on the NA12878 variants. Coverage counts were generated using covcollect with 2kb intervals. MuTect allele counts were processed to remove artifacts based on MAPQ and MuTect default filtering, but were only filtered for het site status if the normal reference sample was derived from NA12878, since the FFPE normals have a separate set of variants. While het site filtering is not essential, since the simulated tumor allele counts are based on the total read depth at the variant depths rather than the individual allele counts, we felt it was appropriate to use the same het site filtering when possible in order to provide HapASeg with a representative number of het sites. The coverage counts were normalized by the average fragment length as described in Coverage processing and passed with the allele counts to the tumor simulation procedure.

The resulting simulated files were passed to HapASeg, and the method was run using the default settings. HapASeg produces two segmentation files: one from the ACDP and another directly from the coverage MCMC (called skip_acdp_segfile). To avoid over-clustered results from the ACDP, we use the skip segmentation files for samples with low purity $(<0.3)$ to compute downstream analyses.

#### HATCHet

We installed HATCHet v1.1.1 from the available github repository in a docker container with its dependencies [42]. We genotyped each variant site in NA12878 using the genotype-snps function using the default parameters. Allele counts and coverage counts were computed using the count-alleles and count-reads functions with default settings, respectively. HATCHet also optionally performs phasing to improve calling accuracy. We enabled phasing on the genotype files for all samples. The phased SNP result files were saved for each sample type for use during copy number calling. The allele count and read count files were passed as inputs to the tumor simulation procedure. The simulated counts were then passed to combine-counts to merge coverage bins and then to cluster-bins to perform the copy number calling. Allelic coverage estimates were extracted from the resulting clustered bins and cluster segments files. We used the cluster estimates assigned to each segment rather than the segment specific estimates, as is presented in the method’s final result plots. These allelic coverage estimates were used for downstream comparisons. Of note, the Richter’s sample CH1022 failed to complete HATCHet due to a segfault during phasing that could not be resolved. All other sample-method pairs ran to completion.

### 5 Miscellaneous calculations

#### Expected length before phase switch

Eagle2 reports a 0.5% error rate [40], which is defined as the number of phase switches required to obtain the true haplotype, normalized by the number of possible switch error positions, which is simply the number of homozygous markers in the individual [64]. Recent studies estimate the average number of homozygous SNPs in an individual to be 404,700 [65], which results in an expected switch error every 14.2Mb.
