# Supplementary material for: Haplotype-aware segmentation with HapASeg increases accuracy of detecting homolog-specific somatic copy number alterations
Source: Genome Biol. 2026 Feb 14;27:83. doi: 10.1186/s13059-026-03971-w (PMC12983724; doi:10.1186/s13059-026-03971-w)
Supplement: Supplementary file 2 — Additional file 2. Supplementary figures. Fig. S1.: HapASeg benchmarking pabel schematic. Fig. S2: AAD calculation schematic. Fig. S3: Fresh frozen WES benchmarking results heatmap. Fig. S4: TCGA matched fresh frozenand FFPE WGS results. Fig. S5: Simulated tumor karyotype summary statistics. [file 13059_2026_3971_MOESM2_ESM.pdf]

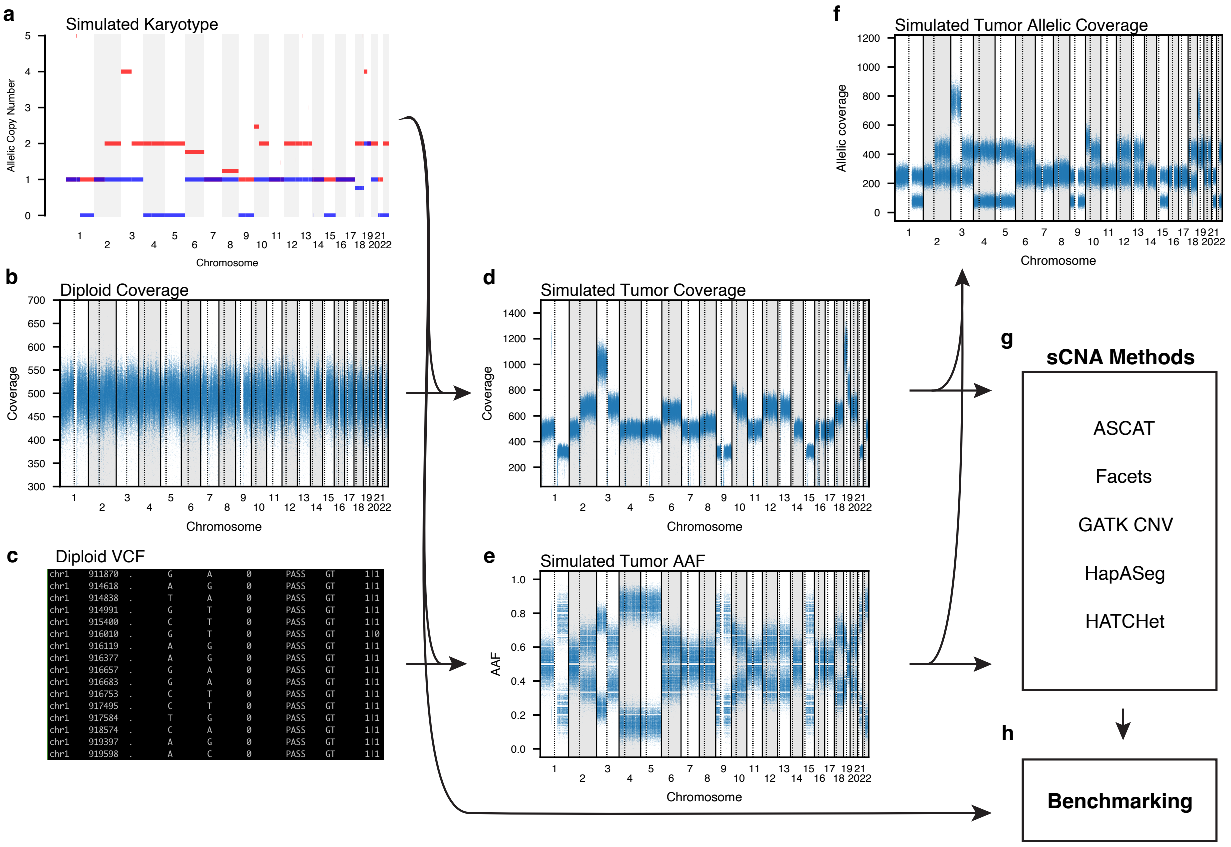

Figure S.1: HapASeg benchmarking panel schematic a, Simulated tumor karyotype is randomly generated with biologically realistic characteristics. **b**, Coverage is computed at 2kb genomic intervals in confidently diploid, normal tissue. **c**, Genotypes and phasing information are calculated for the normal tissue (using Eagle2). **d**, Simulated tumor coverage is generated by scaling the coverage level in the confidently diploid coverage to the TCR level and purity of the simulated karyotype. **e**, Allele counts at heterozygous sites are generated by scaling the allele counts in the diploid tissue by the homolog specific copy number designated by the simulated karyotype. **f**, The simulated TCR is multiplied by the average AAF in each covered bin to compute the simulated allelic coverage for visualization purposes. **g**, State-of-the-art sCNA methods receive the simulated tumor coverage and AAF as input. **h**, The results from the sCNA methods are compared to the simulated karyotype and AAD scores are computed for quantitative benchmarking.

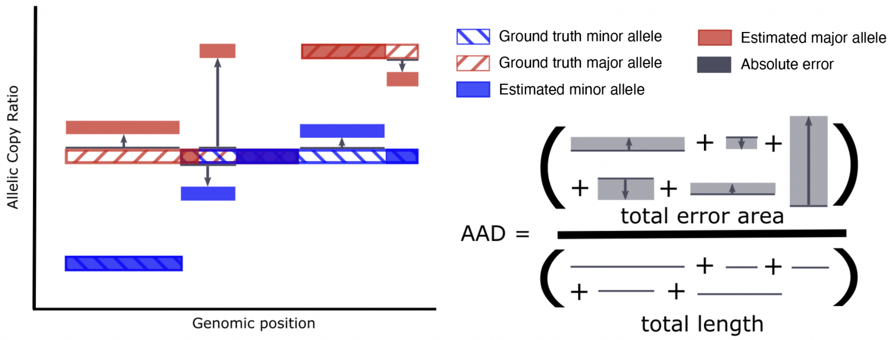

Figure S.2: AAD calculation schematic. The average absolute difference is computed by taking the length-weighted average of the absolute differences between the ground truth copy ratios and the method estimated copy ratio for both alleles.

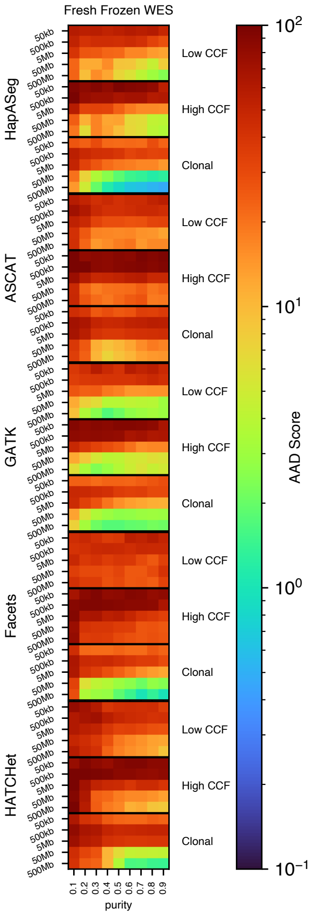

Figure S.3: Fresh frozen WES benchmarking results heatmap. AAD heatmap examines the accuracy of the sCNA methods stratified by purity, ground truth event length and the cancer cell fraction (CCF) of the simulated sCNA segments (clonal: CCF=1, low: CCF $<$ 0.7, high: CCF $\geq$ 0.7).

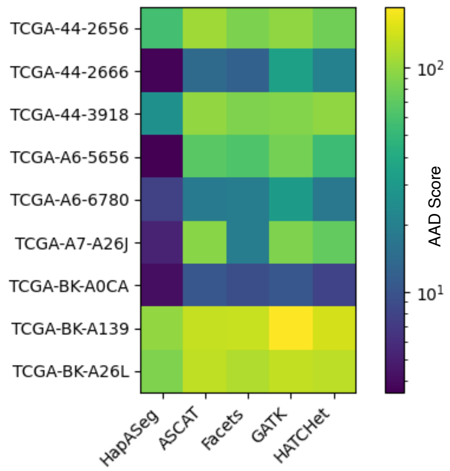

Figure S.4: TCGA matched fresh frozen and FFPE WGS results. The nine cases in TCGA with available fresh frozen and FFPE WGS sequencing from the same tumor sample were processed by the five sCNA calling methods. AAD Scores for the FFPE samples were computed for each method on each sample using the fresh frozen results as ground truth.

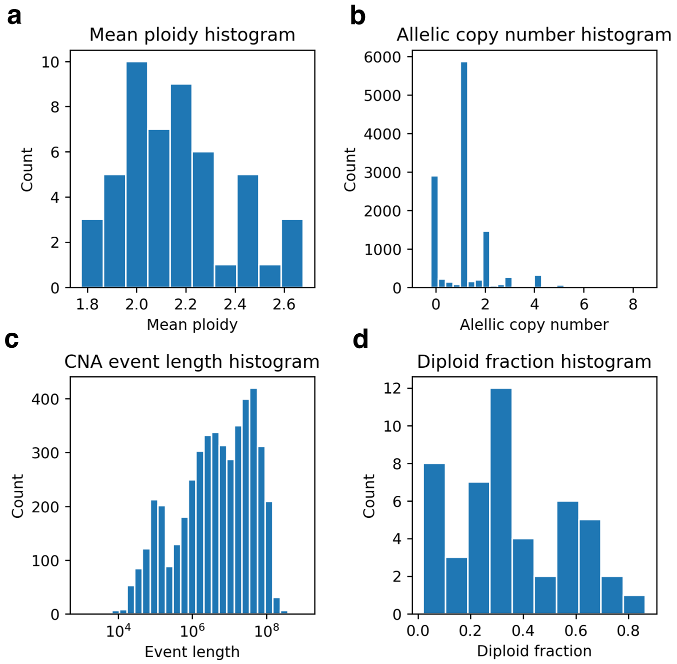

Figure S.5: Simulated tumor karyotype summary statistics. The 50 simulated tumor karyotypes generated for quantitative benchmarking are summarized by their **a.** mean ploidy, **b.** sCNA allelic copy number levels, **c.** sCNA event lengths, and **d.** fraction of the genome with diploid copy number.
